# Rasal1 regulates calcium dependent neuronal maturation by modifying microtubule dynamics

**DOI:** 10.1186/s13578-024-01193-w

**Published:** 2024-01-21

**Authors:** M. H. S. Deurloo, S. Eide, E. Turlova, Q. Li, S. Spijker, H.-S. Sun, A. J. A. Groffen, Z.-P. Feng

**Affiliations:** 1 Department of Physiology, University of Toronto, Toronto, Canada; 2 Department Molecular and Cellular Neurobiology, Neurogenomics and Cognition Research, VU University of Amsterdam, Amsterdam, The Netherlands; 3 Department of Surgery, University of Toronto, Toronto, Canada; 4 Department of Functional Genomics, Center for Neurogenomics and Cognition Research, VU University Amsterdam, Amsterdam, The Netherlands

**Keywords:** RASAL1, Ras signaling, GTPase-activating protein, RasGAP1-like, RAS protein activator-like 1, RapGAP, Rap1

## Abstract

**Background:**

Rasal1 is a Ras GTPase-activating protein which contains C2 domains necessary for dynamic membrane association following intracellular calcium elevation. Membrane-bound Rasal1 inactivates Ras signaling through its RasGAP activity, and through such mechanisms has been implicated in regulating various cellular functions in the context of tumors. Although highly expressed in the brain, the contribution of Rasal1 to neuronal development and function has yet to be explored.

**Results:**

We examined the contributions of Rasal1 to neuronal development in primary culture of hippocampal neurons through modulation of Rasal1 expression using molecular tools. Fixed and live cell imaging demonstrate diffuse expression of Rasal1 throughout the cell soma, dendrites and axon which localizes to the neuronal plasma membrane in response to intracellular calcium fluctuation. Pull-down and co-immunoprecipitation demonstrate direct interaction of Rasal1 with PKC, tubulin, and CaMKII. Consequently, Rasal1 is found to stabilize microtubules, through post-translational modification of tubulin, and accordingly inhibit dendritic outgrowth and branching. Through imaging, molecular, and electrophysiological techniques Rasal1 is shown to promote NMDA-mediated synaptic activity and CaMKII phosphorylation.

**Conclusions:**

Rasal1 functions in two separate roles in neuronal development; calcium regulated neurite outgrowth and the promotion of NMDA receptor-mediated postsynaptic events which may be mediated both by interaction with direct binding partners or calcium-dependent regulation of down-stream pathways. Importantly, the outlined molecular mechanisms of Rasal1 may contribute notably to normal neuronal development and synapse formation.

**Supplementary Information:**

The online version contains supplementary material available at 10.1186/s13578-024-01193-w.

## Background

Rasal1, first described to our knowledge by M. Allen in 1998 [1], is a highly conserved GTPase Activating Protein (GAP) with dual specificity for Ras and Rap [2, 3]. Rasal1 has previously been studied in the context of tumours where it has been found to be down-regulated, implicating it has important cellular activities [4–6]. Rasal1 is highly expressed in the brain, the medulla and in the zona glomerulus of the kidney [1]. However, despite its high expression level in the brain, the role of Rasal1 in neuronal processes remains unexplored.

The structure of Rasal1 gives many clues to its cellular function [1]. The central RasGAP domain of the protein is flanked by two membrane-interacting motifs [7]. The N-terminus features two fully conserved C2 domains [8–10], which share similarity with the Ca^2+^-dependent phospholipid-binding domains of synaptotagmin, protein kinase C and DOC2. The C2A domain of Rasal1 interacts with phosphatidylserine; the C2B domain with phosphoinositides, together encoding a Ca^2+^-dependent membrane association mechanism that is accompanied by activation of RasGAP activity [11]. The C-terminal region contains a pleckstrin homology (PH) domain, which operates as a membrane-binding module in conjunction with the action of the C2 domains [12, 13].

Rasal1 is an interesting candidate specifically in neuronal development for two reasons. Firstly, its responsiveness to intracellular Ca^2+^ oscillations [14]. Intracellular Ca^2+^ signals mediate various cellular processes such as cell proliferation, growth cone motility, synaptic plasticity and neurotransmitter release [15]. Many of these phenomena are influenced by both the frequency and subcellular location of Ca^2+^ oscillations through the workings of Ca^2+^-responsive proteins [16]. For instance, CaMKII decodes the number and frequency of intracellular Ca^2+^ oscillations and in turn utilizes kinase activity to contribute to frequency-dependent forms of synaptic plasticity [17] and neurite outgrowth [18, 19]. Furthermore, the PKC signal transduction pathway regulates the actin cytoskeleton and neurite outgrowth in response to intracellular Ca^2+^ changes [20]. Thus, conceivably as an additional Ca^2+^-sensitive protein, Rasal1 may be implicated in the above neuronal processes.

Secondly, Rasal1 exerts RasGAP activity. Following activation, Ras triggers several pathways involved in cell growth, differentiation and microtubule stabilization [21, 22]. When bound to Raf, Ras initiates the Raf/Mek/ERK/MAPK signalling cascade [21, 22]. Kinases of the extracellular signal-related kinase (ERK) pathway phosphorylate microtubule-associated proteins (MAPs) and regulate microtubule stability and proliferation [23, 24], thereby affecting key factors for neuronal polarization [25]. In addition, Ras activates PI3K and regulates the activity of the Rho family of small G proteins that plays significant roles in remodelling spine morphology, suggesting a tight coordination between the Ras and Rho family [26]. Thus, through Ras activation Rasal1 may play a key role in the activation of these signalling cascades and neuronal processes.

Despite the above considerations, the role of Rasal1 in neuronal differentiation remains unexplored. In this study, we investigate the role of Rasal1 in maturation and synaptic development of mouse hippocampal neurons in vitro. Specifically exploring the involvement of Rasal1 in neurite outgrowth, dendritic branching and synaptic density which may be influenced by Ca^2+^-dependent activation of Rasal1’s RasGAP activity and interaction with direct binding partners.

## Results

### Rasal1 expression in developing hippocampal neurons

**Fig. 1:**
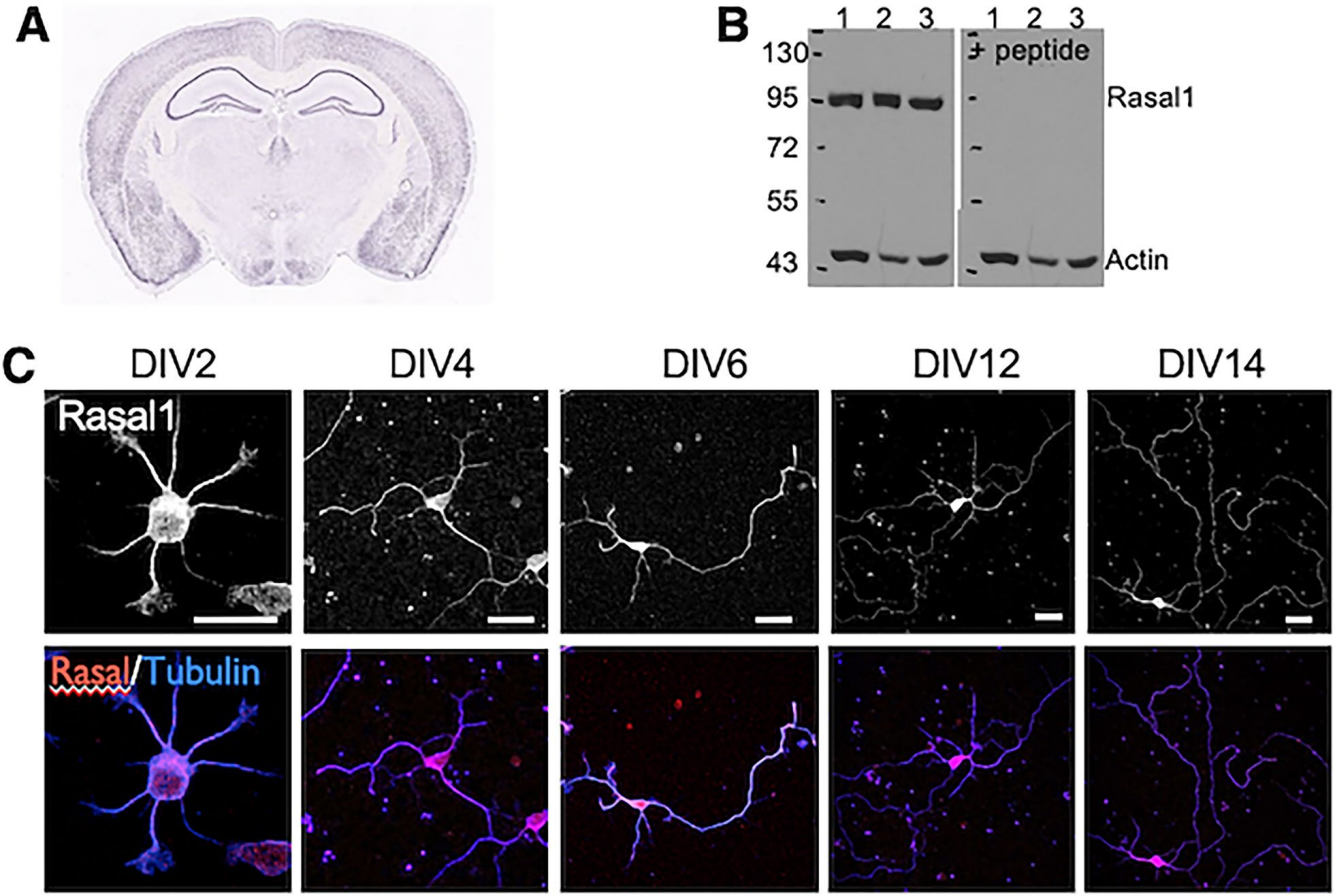
Rasal1 is expressed in the soma and neurites of developing hippocampal neurons. **A** Expression of Rasal1 in coronal section of adult mouse brain. Allen Mouse Brain Atlas, http://mouse.brain-map.org/experiment/show/75774111. **B** Western blot analyses with Rasal1 antibody in the absence (left) and presence (right) of Rasal1 immunizing peptide. Lane 1: mouse hippocampus; lane 2: mouse prefrontal cortex; lane 3: rat hippocampus all at P8. **C** Endogenous Rasal1 immunoreactivity on DIV2, DIV4, DIV6, DIV12 and DIV14 in neurons stained for Rasal1 (red) and Tubulin (blue) and imaged with confocal microscopy. DIV 2 scale bar: 5 μm, DIV 4–10 scale bar: 10 μm

Rasal1 is a Ca^2+^ sensing RasGAP that has not previously been studied in brain development. The Allen Brain Atlas reports broad expression of the Rasal1 gene in the adult murine brain (Fig. 1A). We confirmed Rasal1 expression in specific brain regions using Western blot analysis on samples derived from 8-day old mice and rats (Fig. 1B) which showed a strong single band at the expected molecular mass of 90 kDa in mouse hippocampal tissue (lane 1), mouse prefrontal cortex (lane 2) and rat hippocampal tissue (lane 3). To validate the specificity of the Rasal1 Gen Script antibody, the second blot was incubated with a Rasal1 immunizing peptide (20 µM), which corresponds to the epitope recognized by the antibody. As seen in Fig. 1A the staining was absent in the blot incubated with the peptide demonstrating Rasal1 specificity.

To further evaluate the expression and cellular distribution of Rasal1 protein in developing neurons, we investigated primary cultures from E17-stage mouse hippocampi using immunocytochemical assays. Neurons were stained with Rasal1 antibody on DIV2 to DIV14 (days in vitro) to examine Rasal1 expression from early to late stages of neuronal development in primary culture, during which neurons undergo many developmental processes including neurite outgrowth, branching and synaptogenesis. Confocal imaging shows Rasal1 immunoreactivity in the soma, dendrites and axons of hippocampal neurons from DIV 2 onward (Fig. 1C).

### Rasal1 translocation to the plasma membrane in response to Ca^2+^ in hippocampal neurons

**Fig. 2:**
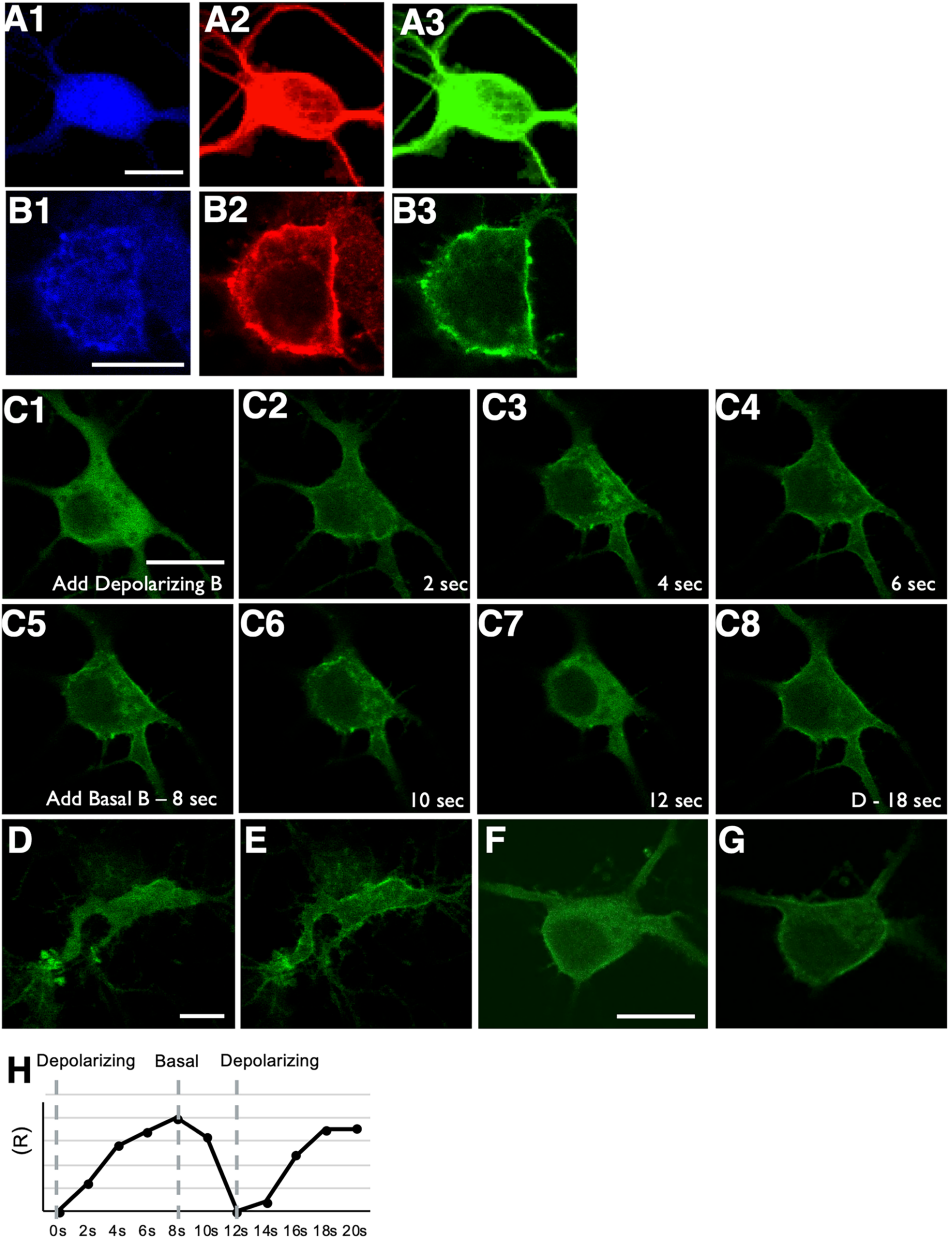
Rasal1 translocates to the plasma membrane in response to Ca^2+^ in hippocampal neurons. **A1-3** Hippocampal neuron expressing Rasal1-EGFP stained to show the distribution of Rasal1-EGFP (green) compared to total Rasal1 immunoreactivity (red) and MAP2 (blue) under basal conditions. Scale bar: 10 µm. **B1-3** Depolarized hippocampal neuron expressing Rasal1-EGFP stained to show Rasal1-EGFP (green) distribution compared to total Rasal1 (red) and MAP2 (blue). **C** Live confocal imaging of neuron expressing Rasal1-EGFP following perfusion of depolarizing buffer (Depolarizing B) **C1-4**. Followed by perfusion with basal buffer (Basal B) at 8 s (C5-7) followed by second perfusion with depolarizing buffer at 18 s (D-18s) (C8). Scale bar: 10 µm. **D**–**G** Rasal1-EGFP live imaging in two additional neurons perfused in basal buffer **D**, **F** and depolarizing buffer (**E**, **G**). Scale bar: 10 µm. **H** Time-dependent change in fluorescence ratio (R) in the membrane after addition of depolarizing buffer, basal buffer at 8 s and depolarizing buffer again at 12 s (addition of buffers indicated by dashed lines). Data presented as percentages of the original fluorescence at 0 s. Mean ± SEM are plotted, n = 20

Since Rasal1 was found to be expressed throughout developing neurons of the hippocampus we next sought to explore the potential functionality of Rasal1 in these cells. Study of Rasal1 in HeLa cells determines that the plasma membrane expression of Rasal1, which is critical to Ras GTPase activity, occurs in response to elevated Ca^2+^ [14]. We hypothesized that neuronal Rasal1 can translocate to the plasma membrane in a Ca^2+^-dependent manner as well. To test this hypothesis, the dynamic changes in localization of a recombinant Rasal1-EGFP fusion protein transfected into the neurons using lipofectamine in response to Ca^2+^ fluctuations were assessed. Endogenous Rasal1 (red) and Rasal1-EGFP (green) proteins had a similar distribution pattern in neurons in basal conditions (Fig. 2A), as well as in depolarized neurons where they showed a similar membrane association (Fig. 2B). Live imaging of Rasal1-EGFP fusion protein (Fig. 2C) showed that perfusion of depolarizing solution induced a rapid plasma membrane association which started after 2 s and was complete after 8 s. The translocation of Rasal1-EGFP was reversible and repeatable up to 5 times in a time frame of 10 min (Fig. 2C1-8). Figure 2D–G show additional examples of Rasal1-EGFP membrane translocation in living neurons, and Fig. 2H shows the time course of membrane translocation ratio (R) of Rasal1 following depolarization with high extracellular KCl (40 mM).

These results confirm that Rasal1 is sensitive to changes in intracellular Ca^2+^ and that resulting reversible plasma membrane association of Rasal1 does occur in neurons as has been observed in other cell types.

### Rasal1 binds to CaMKII, PKC, and α- and ß tubulin

**Fig. 3:**
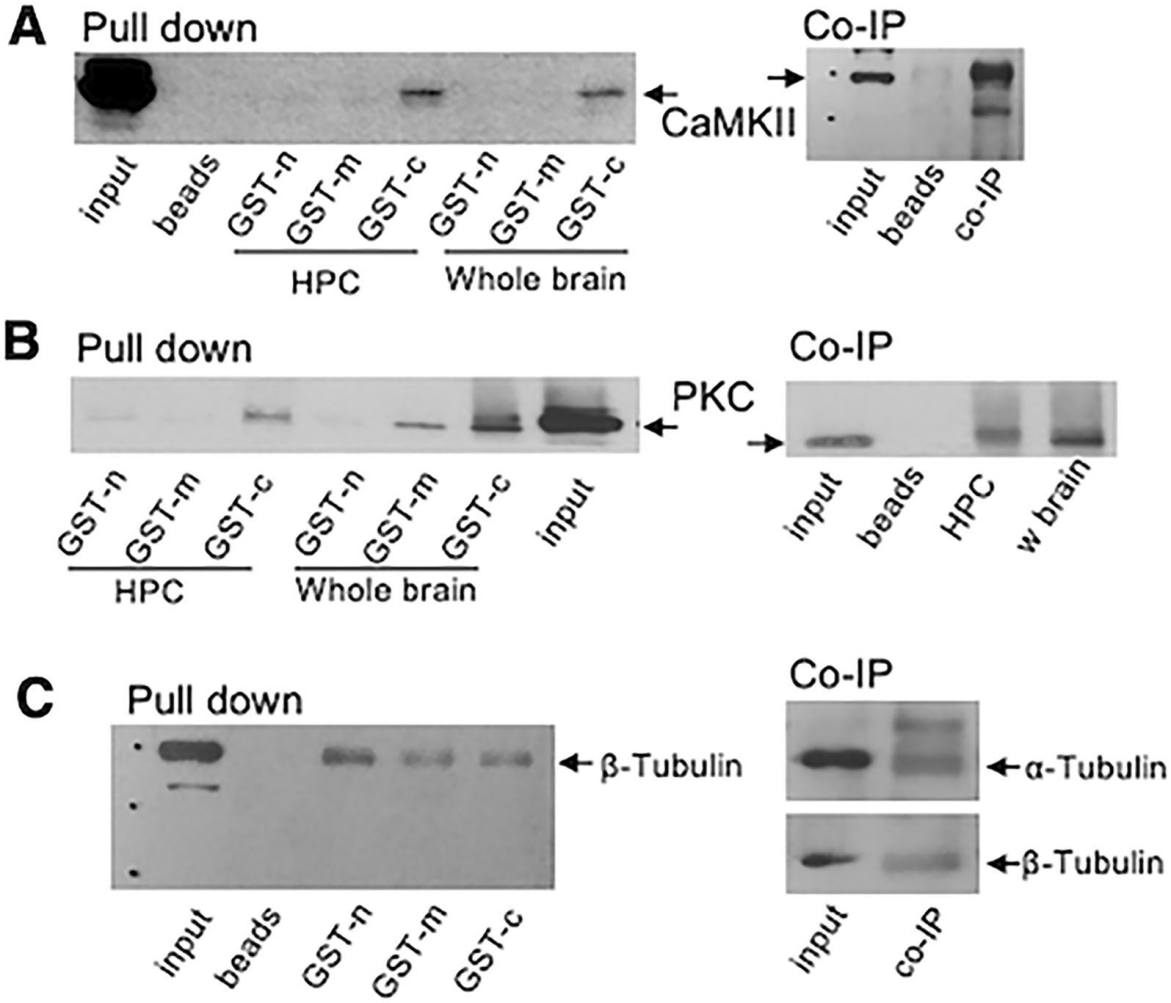
Rasal1 binds to CaMKII, PKC, α- and β-tubulin. Confirmation of suspected Rasal1 binding partners using pull-down assay (n = 3) with GST tagged Rasal1 fragments; N-terminus (GST-n), middle fragment (GST-m) or C-terminal fragment (GST-c) validated by Co-IP. **A** Western blot of pull-down assay (left) and Co-IP (right) of GST-tagged Rasal1 fragments stained for CaMKII in hippocampal and whole brain lysate. **B** Western blot of pull-down assay (left) and Co-IP (right) of GST-tagged Rasal1 fragments stained for PKC in hippocampal and whole brain lysate. **C** Western blot of pull-down assay (left) and Co-IP (right) of GST-tagged Rasal1 fragments stained for β-tubulin in hippocampal and whole brain lysate

To further explore the potential signaling mechanisms of Rasal1 in neurons we carried out a pull-down assay using GST tags on the N-terminus (GST-Rasal1-N), the middle (GST-Rasal1-M) or C-terminus (GST-Rasal1-C) of Rasal1. Pull-down assay identified potential binding partners of Rasal1 to include Ca^2+^-sensing oscillating proteins CaMKII-α and protein kinase C (PKC), as well as the microtubule proteins α- and β-tubulin. CaMKII and PKC were found to be interactors of GST-Rasal1-C, which contains a PH domain. Beta-tubulin was found as an interactor of the GST-Rasal1-N fragment, which contains the tandem C2 domains important for membrane translocation and Rasal1’s function as a RasGAP (Fig. 3A–C).

To confirm, a co-immunoprecipitation using the GST tagged Rasal1-N, Rasal1-M and Rasal1-C was carried out for CaMKII, PKC and β-Tubulin (Fig. 3A–C) using hippocampal and whole brain adult mouse brain tissue. Co-IP using the full-length antibody for Rasal1 was also positive for CaMKII, PKC, and α- and β-tubulin in hippocampal brain tissue. These data confirm CaMKII, PKC and α- and β-tubulin as binding partners of Rasal1.

Taking together developmental expression, the binding partners of Rasal1, and the observed RasGAP activating mechanism of Rasal1 translocation to the plasma membrane in response to intracellular Ca^2+^ in neurons, we set to explore two potential processes of neuronal development that Rasal1 may be implicated in neurite outgrowth and synaptic development.

### Molecular tools for modulating Rasal1 expression in neurons

To investigate its role in neuronal development, we created lentivirus and AAV constructs to either knock down or overexpress Rasal1 in cultured hippocampal neurons. Specifically, pAAV-Rasal1-Flag1-IRES-GFP was used to over-express Rasal1. For knockdown, four different shRNA constructs (pLentilox-Rasal1-shRNA#1 through #4) were tested for their capability to down-regulate Rasal1 expression using a U6 promoter for shRNA expression and a CMV promoter to express EGFP as a marker. A Lenti-control-EGFP was used as an additional negative control. Using confocal microscopy in cultured hippocampal neurons, Rasal1-shRNA reduced Rasal1 expression by 28% and Rasal1-FLAG increased Rasal1 expression by 131% compared to control (Additional file 1: Fig. S1B). EGFP expression could be detected 4 days after transduction with a 30% efficiency (Additional file 1: Fig. S1C), suggesting that the mild effect of the shRNA construct was mostly determined by the transfection percentage. In HEK293 cells transfected with a vector encoding mouse Rasal1-flag, pLentilox-Rasal1-shRNA#3-EGFP was the most effective to knockdown Rasal1 protein from 100 to 54% whereas shRNA#1 caused a reduction to 73% of control. The remaining two shRNA constructs tested were not effective in knocking down Rasal1 expression. In HEK293 cells pAAV-Rasal1-Flag1-IRES-GFP effectively increased Rasal1 expression to 230% (Additional file 1: Fig. S1D, E). Similarly, western blot analysis of Rasal1 protein expression in primary hippocampal neurons (Additional file 1: Fig. S1F, G), demonstrated that pLentilox-Rasal1-shRNA#3 was the most effective knockdown construct, reducing expression to 22% of endogenous Rasal1, and pAAV-Rasal1-Flag1-IRES-GFP (OE1) was most effective for overexpression of Rasal1 with an over 18-fold increase of endogenous expression and were thus chosen for subsequent experiments requiring modulation of neuronal Rasal1 expression. Where applicable, cultures were treated with viral constructs on DIV1 18 h after plating the cells for all experiments.

### Rasal1 inhibits neurite outgrowth

**Fig. 4:**
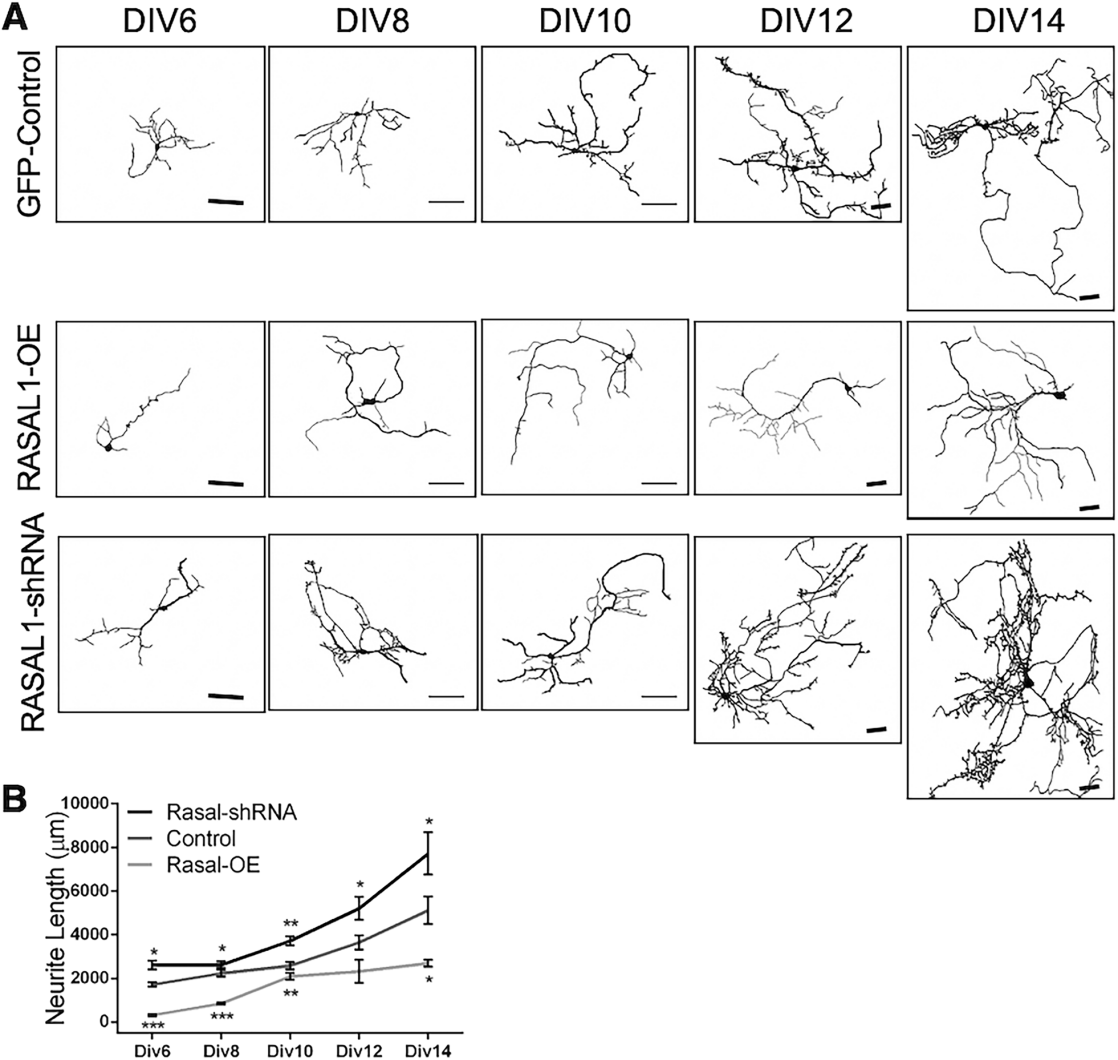
Rasal1 inhibits neurite growth. **A** Neurite tracing using SynD [29] at DIV 6, 8, 10, 12 and 14 of mouse hippocampal neurons transduced with either Rasal1-shRNA-EGFP, Rasal1-OE, or EGFP-control. Scale bar: 50 µm. **B** Neurite length (µm) of EGFP-control, Rasal1-shRNA, and Rasal1-OE expressing mouse hippocampal neurons at DIV 6, 8, 10, 12, and 14. Data pooled from 4 independent experiments (n = 122). Mean ± SEM are plotted, *p < 0.05, **p < 0.01, ***p < 0.001, unpaired student t-test, two tailed

Ras activity is regulated by the balance between activators (guanine exchange factors, GEFs) and inactivators (GTPase activating proteins, GAPs) [27]. Previous study reported the inactivation of Ras is associated with reduction in neurite outgrowth of PC12 neurons [28]. We sought to determine if the RasGAP function of Rasal1 in primary hippocampal neurons may additionally regulate neurite outgrowth. Having validated the molecular tools, we assessed how Rasal1 expression influences neurite outgrowth in cultured mouse hippocampal neurons. Neurites of DIV 6, 8, 10, 12 and 14 hippocampal neurons transduced with either pLenti-Rasal1-Flag1 (Rasal1-OE), Rasal1-shRNA-EGFP (Rasal1-shRNA), or Lenti-control-EGFP (EGFP-control) were traced using SynD (Fig. 4A). Tracings of neurites were used to measure total neurite length per neuron (Fig. 4B). Over-expression of Rasal1 reduced the average total neurite length per neuron compared to EGFP-control, which was significant at DIV 6, 8, 10, and 14 (EGFP-control: Div6 1722 ± 102 µm; Div8 2231 ± 157; Div10 2581 ± 162; Div12 3652 ± 320; Div14 5124 ± 624; vs Rasal1-OE: Div6 320 ± 52; Div8 852 ± 44; Div10 2098 ± 154; Div12 2323 ± 524; Div14 2695 ± 152 (n = 80–120 cells)) (Fig. 4B). To the contrary, Rasal1-shRNA transduced neurons were observed to have significantly greater total neurite length (Div6: 2610 ± 200 µm; Div8: 2620 ± 156; Div10: 3724 ± 213; Div12: 5212 ± 521; Div14: 7721 ± 975; n = 80–120) as compared to controls at all measured time points from DIV 6 to 14 (Fig. 4B).

### Rasal1 inhibits dendrite length and branching

**Fig. 5:**
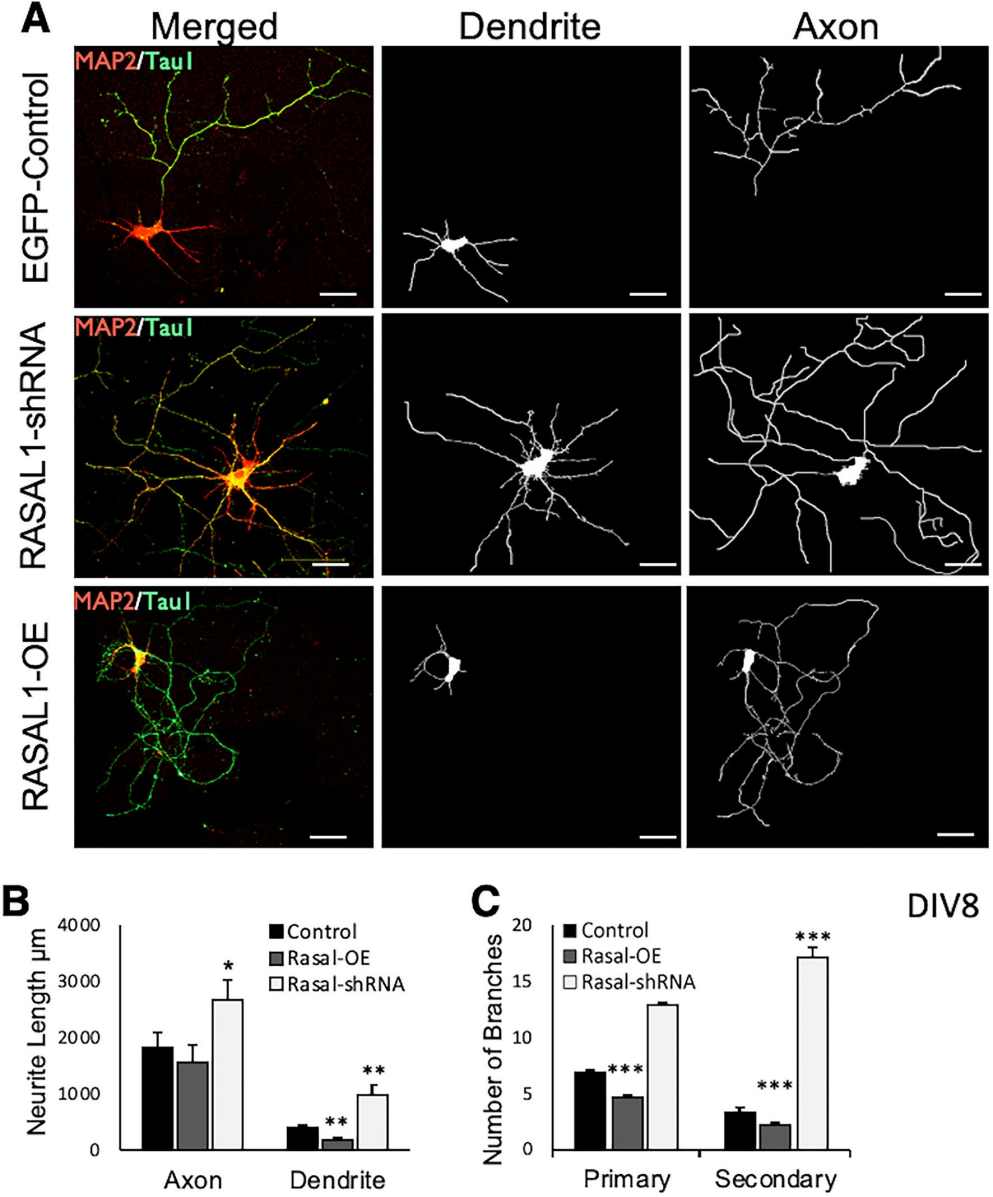
Rasal1 inhibits dendrite length and branching. **A** Representative confocal images of EGFP-control, Rasal1-OE, and Rasal1-shRNA transduced mouse hippocampal neurons stained for the dendritic marker MAP2 (red) and axonal marker Tau1 (green) (left). Tracings of dendrites (middle) and axonal neurites (right) were done using SynD. Scale bar: 20 µm. **B** Total axon and dendrite length (µm) of EGFP-control, Rasal1-OE, and Rasal-shRNA expressing neurons. **C** Number of primary and secondary dendritic branches in neurons expressing EGFP-control, Rasal1-OE, and Rasal1-shRNA. Data information: In **B**, **C** Mean ± SEM are plotted, *p < 0.05, **p < 0.01, ***p < 0.001, unpaired student t-test, two tailed. n = 60 for each group, pooled from 3 repeated experiments

To determine whether the observed effects of Rasal1 expression on total neurite outgrowth was due to specific effects on either axonal or dendritic outgrowth we stained DIV 8 mouse hippocampal neurons with MAP2 and Tau1 antibodies as markers for dendrites and axons respectively. Stained neurons were used to independently trace dendritic and axonal neurites using SynD (Fig. 5A). Comparing dendrite length across groups revealed that over-expression of Rasal1 resulted in significant reductions in dendritic outgrowth relative to controls while Rasal1-shRNA transduced neurons were shown to have significantly longer dendrites (EGFP-control 403.8 ± 35; Rasal1-OE 971.8 ± 15; Rasal1-shRNA 195.9 ± 251, n = 60 (Fig. 5B)). Further, while axonal length was not shown to differ between controls and Rasal1 over-expressing neurons, knockdown of Rasal1 was associated with a significant increase in axonal length (EGFP-control 1857.6 ± 288; Rasal1-OE 1586 ± 302; Rasal1-shRNA 2692 ± 299, n = 60 Fig. 5B).

To further elucidate the way in which Rasal1 influences neurite outgrowth, the effects of Rasal1 on dendritic branching were explored. Rasal1-overexpressing neurons were shown to have significantly less primary and secondary dendritic branches compared to EGFP-controls with greater reductions found in the secondary branches (primary branches: EGFP-control 6.8 ± 0.4; Rasal1-OE 4.7 ± 0.3. secondary branches: EGFP-control 3.3 ± 0.62; Rasal1-OE 2.2 ± 0.1. n = 60 (Fig. 5C)). Comparatively, Rasal1-shRNA transduced neurons were observed to have significantly more secondary dendritic branches (17.25 ± 1.1). However, no significant changes in axonal branching were detected. Taken together, our results suggest that while Rasal1 may play a role in regulating axonal growth, the observed inhibitory effects of Rasal1 on total neurite outgrowth may be attributed to inhibition of dendritic growth and branching to a greater extent.

### Rasal1 increases tubulin detyrosination

**Fig. 6:**
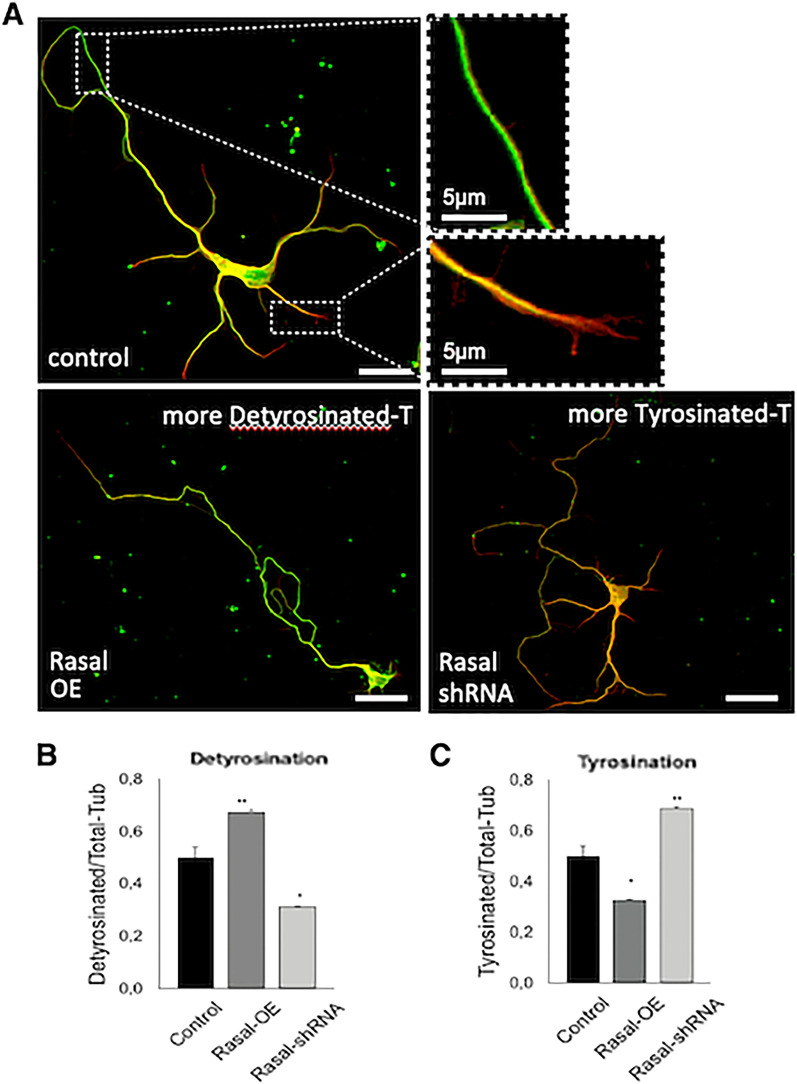
Rasal1 increases tubulin detyrosination. **A** EGFP-control, Rasal1-OE, and Rasal1-shRNA transduced hippocampal neurons stained for tyrosinated tubulin (red) and detyrosinated tubulin (green) at DIV6. Scale bar: 20 μm unless stated otherwise. **B**, **C** Quantification of fluorescence intensity measured in the mid region of the longest neurite of detyrosinated (**B**) and tyrosinated (**C**) tubulin as a proportion of total tubulin. n = 50 for each group, pooled from 5 repeated experiments, mean plus SEM are plotted, *** = p < 0.001, unpaired student t-test, two tailed

A balanced rate of microtubule tyrosination and detyrosination is essential in neuronal differentiation [30]. To test if Rasal1 regulates neurite outgrowth and branching by influencing tubulin dynamics, we compared detyrosinated and tyrosinated tubulin levels in control neurons versus neurons that over-expressed Rasal1 or expressed Rasal1-shRNA. Consistent with the understanding that dendrites require highly tyrosinated tubulin for sustained growth while axons are largely composed of stable detyrosinated tubulin in during development [31, 32], EGFP-control neurons were observed to express tyrosinated tubulin (red) largely in dendritic neurites, with axonal outgrowth comprised of a large pool of detyrosinated tubulin (green) (Fig. 6A). Comparatively, observation of stained neurons over-expressing Rasal1 demonstrated similar presence of detyrosinated tubulin throughout axons but little detyrosinated tubulin in dendritic outgrowths. Inversely, Rasal1-shRNA transduced neurons showed staining of tyrosinated tubulin within dendritic neurites but also throughout axonal outgrowth (Fig. 6A). To quantify levels of tyrosinated and detyrosinated tubulin in neurons, fluorescence intensity was measured in the mid region of the longest neurite. Rasal1-overexpressing neurons showed significantly more detyrosinated tubulin (Fig. 6B, ratio detyrosinated/total-tub, EGFP-control 0.50 ± 0.026; Rasal1-OE 0.67 ± 0.02; Rasal1-shRNA 0.312 ± 0.03). Conversely, Rasal1-shRNA transduced neurons showed significantly more tyrosinated tubulin compared to control (Fig. 6C, ratio tyrosinated/total-tub, EGFP-control 0.499 ± 0.026; Rasal1-OE 0.32 ± 0.002; Rasal1-shRNA 0.687 ± 0.003). This tubulin-detyrosinating activity associated with Rasal1, which is expected to reduce tubulin dynamics, provides one potential explanation for the observed defects in neurite development and specifically may contribute to the observed reduction in dendritic outgrowth which is dependent upon pools of tyrosinated tubulin.

### Rasal1 promotes synapse development and NMDA-mediated synaptic transmission

Beyond neurite outgrowth, Rasal1 expression in developing neurons was thought to play an additional role in regulating synaptic development. Previous study demonstrates SynGAP, an additional neuronally expressed RasGAP, plays a crucial role in synapse development [33]. We thus aimed to explore whether Rasal1 may act in a similar manner.

**Fig. 7:**
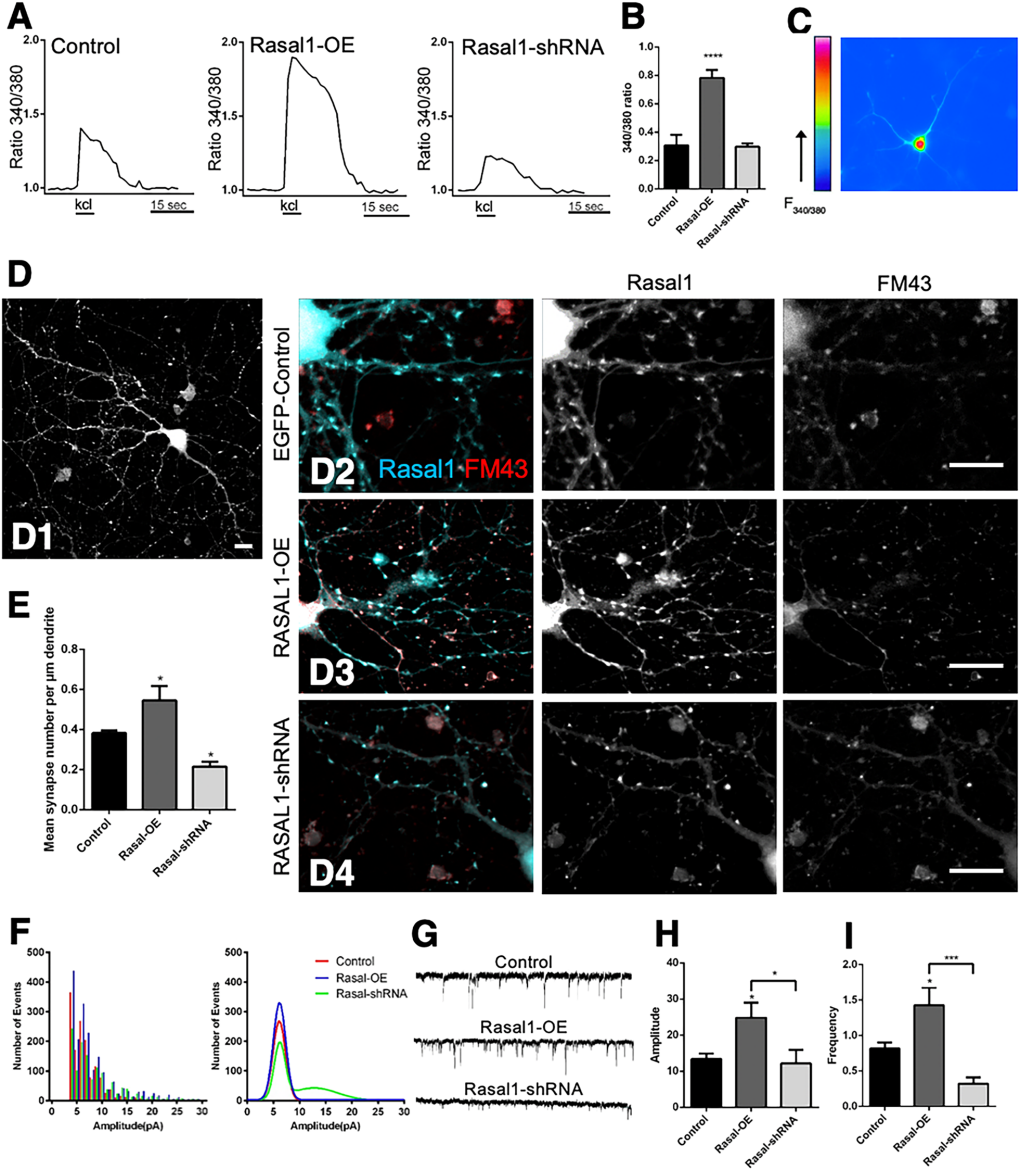
Rasal1 promotes synapse development and NMDA-mediated synaptic transmission. **A** Representative ratiometric measurements of Fura-2 fluorescence, indicative of intracellular Ca^2+^ concentrations, in cultured EGFP-control, Rasal1-OE, and Rasal1-shRNA transduced hippocampal neurons following perfusion with depolarizing buffer. **B** Quantification of Fura-2 ratios in EGFP-control (n-41), Rasal1-OE (n = 47), and Rasal1-shRNA (n = 40) transduced hippocampal neurons following perfusion with depolarizing buffer (40 mM KCl). Mean ± SEM plotted. ****p < 0.0001. **C** Fura-2 color map of loaded neuron. (D1) Punctate expression of endogenous Rasal1 and perinuclear expression in a mature Div16 neuron. (D2-4) Confocal images of synapse morphology stained for FM43 (red) and Rasal1 (cyan) in depolarized EGFP-control, Rasal1-OE, and Rasal1-shRNA transduced hippocampal neurons at DIV 16 day. Scale bar: 10 µM. **E** Quantification of fluorescent FM43 puncta as a measure of functional synapses per µM of dendrite in EGFP-control (n = 45), Rasal1-OE (n = 50), and Rasal1-shRNA (n = 50) transduced hippocampal neurons perfused in depolarizing medium (60 mM KCL). Quantified using SynD software. Data presented as Mean ± SEM. *p < 0.05. **F** The frequency plot (left) and Gaussian distribution (right) of miniature EPSCs (mEPSCs) recorded from EGFP-control, Rasal1-OE, and Rasal1-shRNA transduced hippocampal neurons. **G** Representative traces of isolated NMDA-mediated mEPSCs in EGFP-control, Rasal1-OE, and Rasal1-shRNA transduced hippocampal neurons. **H** NMDA-mediated mEPSC amplitude (pA) of EGFP-control (n = 28), Rasal1-OE (n = 29), and Rasal1-shRNA (n = 19) transduced hippocampal neurons. Mean ± SEM. *p < 0.05. (I) NMDA-mediated mEPSC frequency (Hz) of EGFP-control (n = 28), Rasal1-OE (n = 29), and Rasal1-shRNA (n = 19) transduced hippocampal neurons. Mean ± SEM. *p < 0.05, ***p < 0.001. Data information: In (**B**, **E**, **G**, **H**) data was analyzed using two-tailed unpaired student t-test

We first asked whether Rasal1 affects Ca^2+^ levels in hippocampal neurons in response to depolarization. Fura-2 ratiometric Ca^2+^ signals were compared in control cells, neurons overexpressing Rasal1 and neurons expressing Rasal1-shRNA, as described above. Figure 7A shows representative Fura-2 traces of the 3 groups. After perfusion with 40 mM KCl control neurons showed 340/380 ratios of 0.306 ± 0.07, n = 41. Ca^2+^ levels were significantly increased in Rasal1-OE neurons (Fura-2 340/380 ratio of 0.78 ± 0.05, n = 47 P < 0.00001 compared to control). Ca^2+^ levels in Rasal1-shRNA transduced neurons were not significantly reduced compared to EGFP-control (Fura-2 ratio 0.29 ± 0.022, n = 40; Fig. 7B).

Synapse development requires optimal Ca^2+^ levels [34]. We asked if Rasal1 would play a role in synapse development in hippocampal neuronal culture. Figure 7D1 shows a punctate expression of endogenous Rasal1 and somatic expression in a mature DIV16 neuron. Cellular Rasal1 levels were manipulated by either overexpression or knockdown, and the synapse morphology after 16 days in culture was observed. To this end, neurons were depolarized in 60 mM KCl to allow endocytic uptake of the lipophilic dye FM1-43, a well-established method to label active synapses [35]. Specifically, synaptic density was compared between transduced hippocampal cultures with EGFP-control, Rasal1-OE and Rasal1-shRNA (Fig. 7D2,3,4). Synapse puncta were counted using SynD software per µm dendrite. Neurons overexpressing Rasal1 had significantly more puncta 0.54/µm ± 0.014, n = 50, compared to control 0.38/µm ± 0.014, n = 45, *P* < 0.01. Consistently, Rasal1-shRNA neurons had significantly less puncta compared to control (0.21/µm ± 0.025, n = 50, *P* < 0.01) (Fig. 7E). For all 3 groups, 90% of the fluorescent puncta were also positive for red FM43 dye. Thus, Rasal1 promotes morphological development of functional synapses.

We then investigated if the increased synapses in Rasal1-overexpressing cells enhance synaptic transmission. Specifically, we compared the miniature EPSCs (mEPSCs) recorded from the 3 neuronal groups. The recordings were carried out in the presence of 0.2 μM tetrodotoxin (TTX, Tocris), 200 μM CdCl2, 10 μM bicuculline (BCC, Sigma) and 20 μM CNQX (Tocris). The Gaussian and binomial distribution show an increased frequency of mEPCSs in Rasal1-OE neurons compared to EGFP-control, and a reduced mEPSCs frequency in Rasal1-shRNA neurons compared to EGFP-control (Fig. 7F, G). When we isolated specific receptors with several receptor blockers, we found that isolated NMDAR-mediated mEPSCs were specifically affected. Example NMDAR-mEPSCs are shown in Fig. 7H. NMDA-mediated mEPSC amplitude was significantly increased in Rasal1-OE neurons 24.8 ± 4.16 pA, n = 29 compared to EGFP-control 13.4 ± 1.5 pA, n = 28, while Rasal1-shRNA neurons were not significantly changed; 12.2 ± 3.8 pA n = 19 (Fig. 7I). NMDA-mediated mEPSC frequency was also significantly increased in Rasal1-OE neurons; 1.4 ± 0.2 Hz, compared to EGFP-control; 0.81 ± 0.08 Hz. mEPSC frequency appeared smaller in Rasal1-shRNA neurons (0.32 ± 0.08 Hz) but was not found to be significantly different from the control group. Together our data show that Rasal1 promotes synapse development and NMDA-mediated synaptic transmission.

### Rasal1 promotes CaMKII phosphorylation

**Fig. 8:**
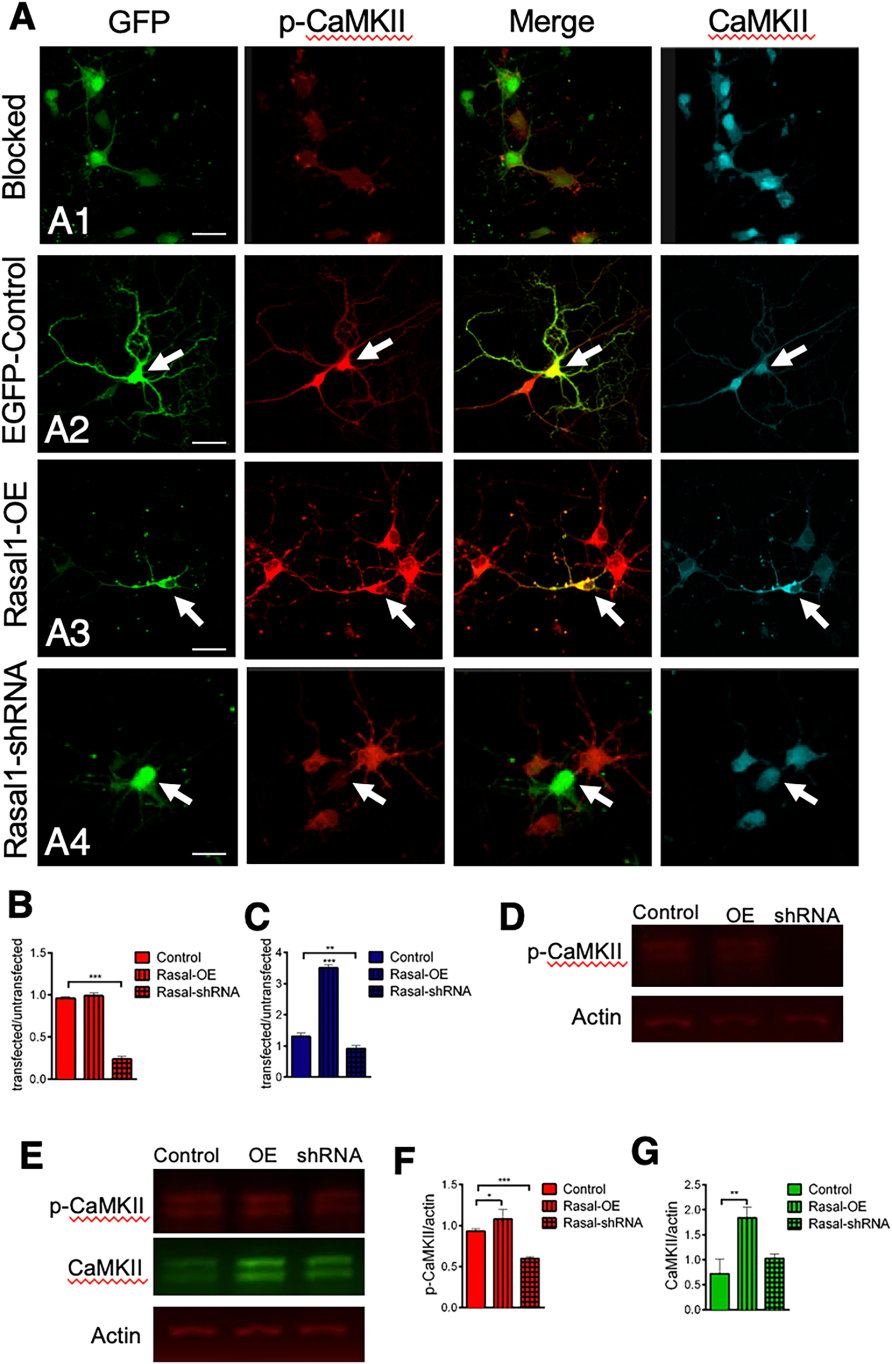
Rasal1 promotes CaMKII phosphorylation. **A** Representative confocal images of blocked control hippocampal neurons treated with TTX, CNQX and APV for at least 8 h to block CaMKII phosphorylation (**A1**), EGFP-control hippocampal neurons treated for 5 min with 10 µM glycine to acutely induce CaMKII phosphorylation (**A2**), glycine (10 µM) treated Rasal1-OE transduced hippocampal neurons (**A3**) and glycine (10 µM) treated Rasal1-shRNA transduced hippocampal neurons (**A4**) stained for phosphorylated (red) or total CaMKII (cyan). Transfected neurons (green) indicated by arrows were compared to neighboring non-transfected neurons. Scale bar: 20 µm. **B** Quantification of the ratio of phosphorylated CaMKII fluorescence intensity of transfected /non-transfected EGFP-control (n = 20), Rasal1-OE (n = 25), and Rasal1-shRNA (n = 25) transduced hippocampal neurons. **C** Quantification of total CaMKII levels in EGFP-control (n = 20), Rasal1-OE (n = 25), and Rasal1-shRNA (n = 25) transduced hippocampal neurons as ratio of fluorescence intensity in transfected/non-transfected neurons. **D** Fluorescent western blot of p-CaMKII (red) of glycine (10 µM) treated EGFP-control, Rasal1-OE, and Rasal1-shRNA transduced hippocampal neurons. **E** Fluorescent western blot of CaMKII (green) and p-CaMKII (red) of glycine (10 µM) treated EGFP-control, Rasal1-OE, and Rasal1-shRNA transduced hippocampal neurons. **F** Quantification of p-CaMKII/actin levels in fluorescent western blot across of glycine (10 µM) treated EGFP-control, Rasal1-OE, and Rasal1-shRNA transduced hippocampal neurons (n = 8). **G** Quantification of total CaMKII/actin levels in fluorescent western blot across of glycine (10 µM) treated EGFP-control, Rasal1-OE, and Rasal1-shRNA transduced hippocampal neurons. (n = 8). Data information: In (**B**, **C**, **F**, **G**) *p < 0.05, **p < 0.01, ***p < 0.001, two-tailed unpaired student t-test

Since Rasal1 was shown to be associated with elevated NMDAR activity, we next explored whether CaMKII activation was additionally regulated by Rasal1 expression as CaMKII activation is known to occur as a result of NMDAR activity (Giese et al. 1998; Bayer et al. 2001). Therefore, investigating the possibility that Rasal1 indirectly activates CaMKII and thereby contributes to NMDA-dependent synaptic development. To do so we used an antibody that specifically detects phosphorylated (i.e. activated) CaMKII (p-CaMKII). To observe the maximal range of activation of CaMKII it was necessary to inhibit spontaneous synaptic transmission before activation. Cultures were treated with TTX, CNQX and APV for at least 8 h (blocked neurons). Under these conditions, the neurons showed a very low level of p-CaMKII immunofluorescence and total CaMKII fluorescence level was stable in all groups (Fig. 8A1). Alternatively, the neurons were first blocked and subsequently activated acutely by applying media containing high glycine for 5 min to facilitate CaMKII activation (activated neurons, Fig. 8A2-4). In Fig. 8A2 activation of control neurons facilitates higher levels of phosphorylated CaMKII (red) confirming that our protocol activated CaMKII.

To test the possibility that Rasal1 enhances CaMKII activation, we compared phosphorylated CaMKII immunoreactivity in neurons expressing EGFP-control, Rasal1-OE and Rasal1-shRNA to neighboring untransfected neurons in activated cultures. The ratio of fluorescence intensity of transfected/untransfected neurons was taken. Rasal1 overexpression did not significantly increase the level of phosphorylated CaMKII compared to EGFP-control (Fig. 8B, EGFP-control ratio: 0.96 ± 0.02 vs Rasal1-OE ratio: 0.98 ± 0.03, n = 20–25). Immunofluorescence in Rasal1-shRNA neurons drastically decreased p-CaMKII immunoreactivity (ratio 0.23 ± 0.03, n = 25; Fig. 8B and A4). Total CaMKII levels in Fig. 8C significantly increased in Rasal1-OE neurons (3.5 ± 0.09) compared to EGFP-control (1.3 ± 0.1, p < 0.0001). Total CaMKII fluorescence also significantly decreased compared to control in Rasal1-shRNA transfected neurons (0.92 ± 0.09, n = 20–25, p < 0.001). To confirm these interesting data, we carried out fluorescent Western blot analysis of p-CaMKII (red) (Fig. 8D), and p-CaMKII (red) and CaMKII (green) on the same blot (Fig. 8E). The hippocampal neurons were treated identically as in the immunofluorescence experiment in Fig. 8A–C. Receptor activation increased p-CaMKII expression in all conditions (Fig. 8E) compared to blocked neurons (Fig. 8D) which shows that our activation protocol works in Western blot. p-CaMKII levels were significantly increased in Rasal1-OE samples (1.07 ± 0.12) compared to control (0.93 ± 0.03, n = 8, p < 0.01) (Fig. 8F). In Rasal1-shRNA transduced neurons, p-CaMKII levels were significantly decreased (0.59 ± 0.02) compared to control (p < 0.0001). Total CaMKII levels were significantly higher in Rasal1-OE transduced neurons compared to EGFP-control while Rasal1-shRNA transduced neurons were not significantly different (EGFP-control: 0.72 ± 0.29; Rasal1-OE: 1.83 ± 0.21; Rasal1-shRNA: 1.02 ± 0.08, n = 8) (Fig. 8G). Our findings using Western blot analysis suggests a strong effect of Rasal1 on CaMKII activity, considering that only 40% of the neurons were transfected with the Rasal1-OE or Rasal1-shRNA constructs (Additional file 2: Fig. S2).

## Discussion

We provide the first evidence, to our knowledge, indicating the role of Rasal1 in neurite outgrowth and synaptic development in developing hippocampal neurons. Rasal1 was found to be highly expressed within the hippocampus with diffuse expression across the soma, axon and dendrites of primary hippocampal neurons. Rasal1 expression is found to be linked to both inhibition of dendritic growth and branching as a result of elevations in microtubule detyrosination and to synaptic development through elevations in NMDAR activity.

Overexpression of Rasal1 was found to predominately inhibit dendritic outgrowth and branching, whereas knockdown increased neurite outgrowth and dendritic branching. The observed regulation of neurite outgrowth by Rasal1 may be a feature of observed elevated tubulin detyrosination and thus subsequent reduction in microtubule dynamics, specifically in dendritic neurites which have previously been reported to rely on the dynamic state of tyrosinated tubulin for outgrowth in developing neurons [31, 32]. The tyrosination state of tubulin is a significant factor in microtubule dynamics, with tyrosinated tubulin supporting highly dynamic microtubules and detyrtosinated tubulin associated with stabilized microtubules [36]. Rasal1 expression was shown to correlate with significant elevations in total detyrosinated tubulin within hippocampal neurons, with observed increases in detyrosinated tubulin and reductions in tyrosinated tubulin specifically in dendritic outgrowths compared to controls. Previous study demonstrates that while developing axons express greater amounts of stable detyrosinated tubulin, developing dendrites require higher levels of dynamic tyrosinated tubulin to support outgrowth and branching [31, 32]. With further evidence suggesting that the absence of tyrosinated tubulin alters the morphology and path-finding ability of developing growth cones within dendritic processes [37–39]. Thus, providing a potential explanation as to why Rasal1 expression associated with greater detyrosinated tubulin was observed to reduce dendritic length and branching whereas Rasal1 knock-down, associated with greater tyrosinated tubulin, was observed to increase dendritic outgrowth and branching. However, the mechanisms bridging Rasal1 and tubulin tyrosination states is currently not understood. Additionally, the ability of Rasal1 to mediate neurite outgrowth through other post-translational tubulin modifications, such as acetylation, remain to be investigated.

The observed outgrowth events may also be attributed to both Rasal1’s RasGAP function as well as direct interaction with binding partners. To function as a RasGAP and reduce active Ras [14] Rasal1 must associate with the plasma membrane. The reversible Ca^2+^-induced translocation, likely driven by the conserved C2 domains, provides such an activation mechanism [8–10]. As observed in the current study, neuronal Rasal1 reversibly translocates to the plasma membrane in response to Ca^2+^ elevations allowing for activation of RasGAP activity and inhibition of Ras. Ras inhibition by other GTPase-activating proteins has been previously demonstrated to result in similar reduction in neurite outgrowth in rat PC12 neurons [28], most likely through inhibition of the down-stream MAPK–ERK–Kinase cascade [21, 22, 40]. Activation of ERK by Ras is important for dendritic protein synthesis and functions as a supersensitive Ca^2+^-dependent threshold detector for neural activity in dendrites [41]. As our study demonstrated successful knockdown of Rasal1 expression in neurons using lentiviral particles, it is expected that consequently a reduction of RasGAP activity through reduced Rasal1 would lead to less inhibition of active Ras and down-stream signaling proteins which could explain the observed increase in neurite outgrowth and branching. Conversely, the observed inhibition of neurite outgrowth as a result of Rasal1 over-expression may be explained through increased inhibition of Ras.

Further, it is suggested that Rasal1’s interaction with identified binding partners, CaMKII, PKC, and tubulin, may also modulate neurite outgrowth by modifying microtubule dynamics. Microtubules are highly dynamic, undergoing cycles of polymerization and rapid depolymerization [30] regulated by calcium [42]. Axon pathfinding requires different ranges of high and low Ca^2+^ levels that are needed for optimal neurite outgrowth [43]. Activated by asymmetric activation of Ca^2+^ channels in the plasma membrane or internal stores, low Ca^2+^ levels lead to collapse of filopodia [15] while high Ca^2+^ levels cause local stabilization [44]. High Ca^2+^ regulates MAP1B phosphorylation resulting in decreased tubulin binding, favoring disassembly of microtubules [45]. In addition, Ca^2+^-bound calmodulin binds to microtubule-associated proteins MAP2 and Tau-1 and competes for association with tubulin, inhibiting microtubule stabilization [46]. Direct binding of Rasal1 to tubulin may act as a similar obstacle to the cycling of microtubule polymerization in high Ca^2+^ conditions. Moreover, High [Ca^2+^]_i_ elevations activate CaMKII, leading to reorganization of the actin cytoskeleton and regulation of neurite outgrowth [47]. Activation of CaMKII is dependent on calmodulin and Ca^2+^ binding. It is possible that CaMKII keeps calmodulin in close proximity to Rasal1 as we have identified CaMKII as one of Rasal1’s binding partners, along with PKC. With CaMKII, PKC and neuromodulin binding to the PH-domain of Rasal1, which acts as the [Ca^2+^]_i_ oscillation sensor, it is possible that Rasal1, CaMKII, PKC, calmodulin and neuromodulin all work together in a Ca^2+^ decoding complex facilitating membrane translocation and binding. This complex with Rasal1 could functionally be involved in neurite outgrowth, influencing the assembly and disassembly of microtubules.

We further identified Rasal1 as a potential contributor to synaptic development in developing hippocampal neurons. Rasal1 expression was found to be associated with significantly greater influxes of Ca^2+^ following depolarization as well as elevations in frequency and amplitude of mESPCs which were attributed to NMDAR activity. Additionally, Rasal1 expression was associated with subsequently greater elevations in activated CaMKII which is reasonably assumed to be the result of observed elevations in NMDAR activity [48, 49]. Following activation, CaMKII regulates trafficking of AMPA receptors to the postsynaptic density strengthening the postsynaptic response [50]. Altogether, supporting the observed increases in synaptic densities observed in hippocampal neurons over-expressing Rasal1. Interestingly, Rasal1 was found to be a direct binding partner of CaMKII in the present analysis. Although the functionality of this interaction was not explored, it is suspected that similar to CaMKII binding of SynGAP within synapses following NMDA-mediated activation, active CaMKII binds Rasal1 to inactivate Rasal1’s RasGAP activity and sequester the GTPase-activating protein away from synapses in efforts to release Ras and downstream signaling proteins from inhibition [51]. The timed activation of Ras would allow for the activation of ERK-dependent signaling cascades that promote AMPA insertion into the developing synapses, promoting synaptic maturation [52–54]. Suggesting a timed role of Rasal1 in synaptic development wherein Rasal1 present in synapses is activated following depolarization promoting NMDAR activation which subsequently increases CaMKII activity. Following activation CaMKII binds to Rasal1, inactivating Rasal1’s inhibitory effect on Ras, the latter is then free to participate in signaling cascades that promote AMPA insertion into synapses. However, further research is required to explore this theory and should look into the timed expression of Rasal1 in synapses before and after NMDAR activation and the long-term effects of Rasal1 expression on AMPAR expression and insertion.

The mechanism bridging Rasal1 and increased NMDAR activity was not explored in the present analysis and should be considered as an area of interest. A previous study reported that PKC modulates NMDAR trafficking and gating, particularly linking PKC to the rapid delivery and activation of NMDA receptors in hippocampal neurons [55]. This finding suggests a possible mechanism that Rasal1 might directly bind to and sequester PKC at synapses. Such an interaction could facilitate the rapid insertion and activation of NMDARs. Subsequently, these NMDARs may be inhibited and removed from the synapse by CaMKII activated by NMDAR. This sequence of events could then create a conducive environment for the Ras-mediated insertion of AMPA receptors.

Future work should explore the contribution of Rasal1 to physiological processes of learning and memory through the presently identified contributions of Rasal1 to NMDA-mediated synaptic development and interactions with direct binding partners in mature neurons. As well as Rasal1’s role in learning and memory deficits coinciding with neurological disorders. Rasal1 has been shown as one of the earliest deregulated proteins in hippocampus in Alzheimer’s disease [56]. Additionally, it has been shown deregulated in other disorders involving cognitive impairments, such as Huntington’s disease [57] and Parkinson’s disease [58]. This suggests Rasal1 may play a role in abhorrent synaptic function and neuronal degeneration, potentially through interaction with microtubule networks. These findings position Rasal1 not only as a novel protein of interest in neuronal development but also in neurodegeneration.

## Conclusions

**Fig. 9:**
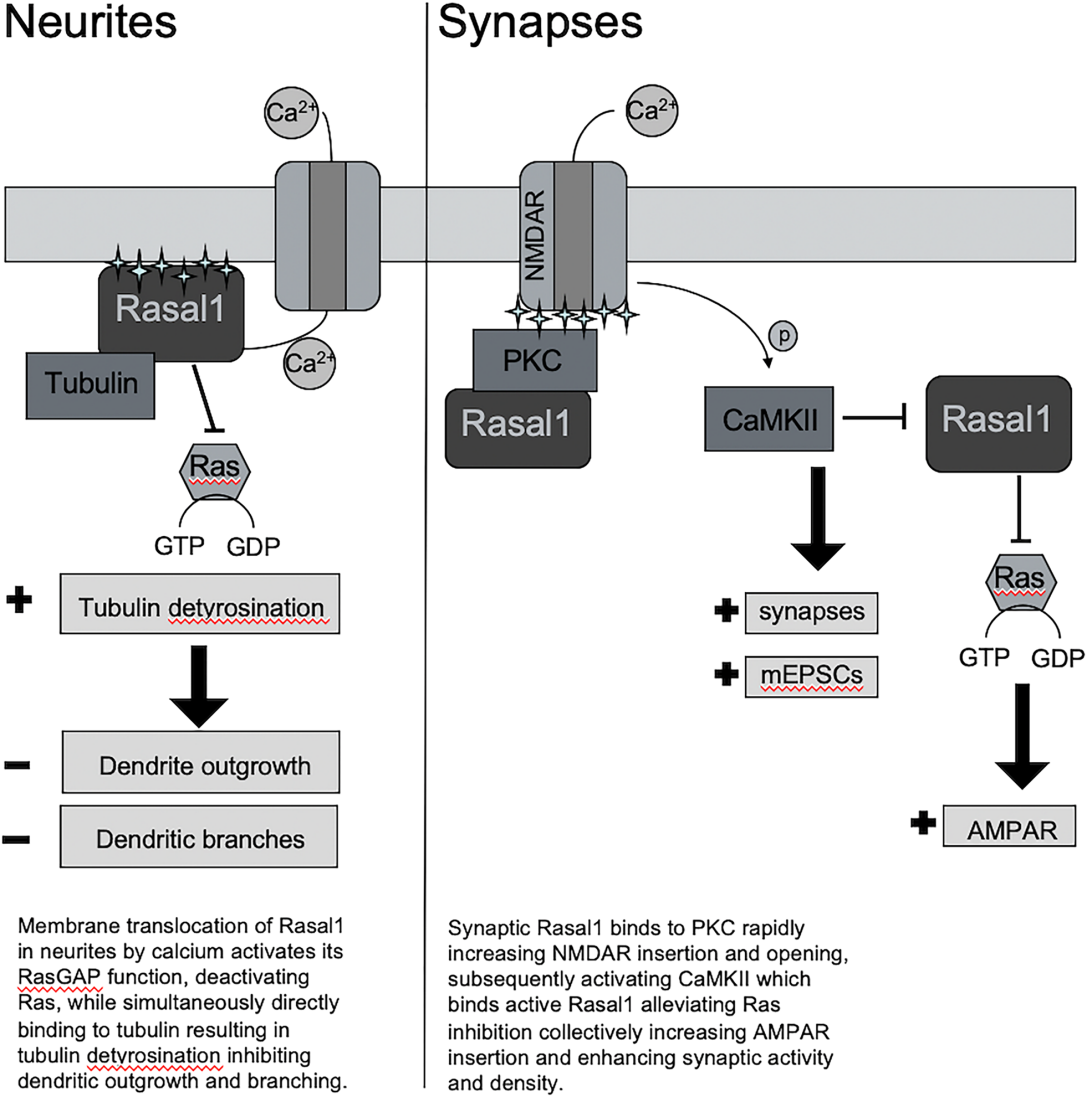
Working model of proposed mechanistic activities of Rasal1 in neurites and synapses

Taken together, our work explores the unknown functions of neuronal Rasal1, supporting two roles for Rasal1 in neuronal development: (1) Inhibition of neurite outgrowth through detyrosination of dendritic microtubules, and (2) strengthening synaptic transmission through elevated NMDAR activity. The current proposed working model of Rasal1 in neurites and synapses is illustrated (Fig. 9).

## Materials and methods

### Animal and dissociated cultures

Embryonic hippocampal cultures were prepared from embryonic day 17–18 CD1 mice following the published protocol [59] with modifications. All procedures were performed in accordance with animal welfare guidelines at the University of Toronto and were approved by the institutional animal care and use committee.

Dissected hippocampi were digested with 0.025% Trypsin/EDTA at 37 °C in HBSS (Hanks balanced salt solution; Sigma, St. Louis, MO, U.S.A) for 15 min and washed with pre-warmed medium to stop digestion. Cells were triturated approximately 10 times with a 1000 μL tip. Neurons were centrifuged at 1000 rpm for 5 min, supernatant was removed and cells were re-suspended in culture media. Cell density was determined using an Improved Neubauer hemocytometer and low-density cultures were plated on Poly-D-Lysine (0.1 mg/ml Sigma) coated glass coverslips (18 mm #1.5, Warner Instruments) at 25,000 neurons/cm^2^. Hippocampal neurons were cultured using a spatially separated ring of cortical neurons for neurotrophic support following the method published previously [60]. Neurons were plated in Neurobasal medium (Invitrogen, Carlsbad, CA, U.S.A.) with 2% B27 (Invitrogen), 1 × Pen/Strep (Gibco), and 2 mM GlutaMAX (Invitrogen) at 37C with 5% CO_2_.

### Transduction of hippocampal neurons

The small hairpin sequence shRNA targeting Rasal1 was packaged in a pLentilox vector with EGFP and CMV promotor. Target sequence of the shRNA#3 was gaagacccgctttccacac. Full-length Rasal1 (Genbank number ID:19415) was packaged in an AAV vector with FLAG tag IRES-GFP. Two controls were either packaged with scrambled siRNA or with EGFP alone. Cultures were treated at Div1, 18 h after plating the cells. The vectors transduced neurons with near 40% efficiency as determined by EGFP expression, which could be detected as early as Div4.

### Immunocytochemistry

For the analysis of neurite outgrowth and branching cultures were fixed at the indicated time points with pre-warmed 4% paraformaldehyde/4% sucrose in PBS for 20 min at RT, and washed 3 × with PBS. Cells were permeabilized with 0.1% Triton X-100 in PBS for 20 min at RT. Antibody solution consisted of 2% bovine serum albumin (BSA), 2% fetal bovine serum (FBS), and 0.2% fish gelatin in PBS. Cells were incubated with primary antibodies in antibody solution for either 2 h at RT or overnight at 4 °C. After the secondary antibody incubation, cells were washed 3 × with PBS. Coverslips were mounted on slides with ProLong Gold (Invitrogen) and dried in the dark at RT.

### Antibodies

We compared several Rasal1 antibodies; Rasal1 sc-68771 Santa Cruz, Rasal1 NBP1-32776 Novus Biologicals and Rasal1 from GenScript (Piscataway, NJ). The GenScript antibody proved to be highly specific and was used in all experiments. Anti-alpha-tubulin (mouse monoclonal, 1:1000, Sigma, #T-5168), anti-beta-tubulin (1:1000, Sigma, #T0198) anti-MAP2 (chicken polyclonal, 1:500, Millipore, AB15452), anti-Tau1 (mouse monoclonal, 1:1000, Chemicon, #MAB3420), anti-Flag (mouse monoclonal, 1:10.000, Sigma Aldrich, F3165), anti-Rasal1 (rabbit polyclonal, 1:500, GenScript), anti-Tyrosinated Tubulin (rat monoclonal, 1:40.000, Abcam, Ab6160), anti-detyrosinated tubulin (rabbit polyclonal, 1:200, Millipore, #AB3201,) and anti-NeuN (mouse monoclonal, 1:200, Millipore, #MAB377, Darmstadt, Germany), anti-CaMKII (mouse monoclonal, Santa Cruz, sc-5306), and anti-pCaMKII (rabbit polyclonal, Santa Cruz, sc-12886). Secondary antibodies: anti-mouse, rat and rabbit, alexa fluor 405, 488, 568 and 688 (1:500, Molecular Probes).

### Western blotting

The procedures of Western blot were carried out as previously described [61]. Protein samples were prepared in RIPA buffer and protein concentrations were determined using the Bio-Rad Protein Assay reagent (Bio-Rad, Hercules, CA). Equal amounts of protein samples (25 µg) were separated in a 10% SDS-PAGE gel and transferred to a nitrocellulose membrane (#66485; PALL Life Sciences) via semi-dry transfer (350 mA, 90 min). Blots were blocked with 5% skim milk in tris-buffered saline with 0.1% tween-20 (TBST), and incubated with primary at 4 ºC overnight and with corresponding horseradish peroxidise (HRP) and then corresponding secondary antibodies at room temperature. Protein signals of interest were visualized using enhanced chemiluminescent reagents (PerkinElmer, Mass, USA) and analyzed by exposure to film (HyBlot CL, NJ, USA). Densitometry was employed to quantify proteins of interest using the gel analyzer function of ImageJ (ver. 1.46a; National Institutes of Health, USA).

### Image acquisition

Images were captured with a Leica TCS LS confocal laser-scanning microscope (Heidelberg, Germany; Leica confocal software, version 2.5; Leica Microsystems) with either 40x (NA 1.25) or 63x (NA 1.32) oil-immersion lenses, and 488, 543 and 633 lasers, as described previously [61] or with a Carl Zeiss Confocal Laser Scanning Microscope LSM700 with either 63 × DIC (NA 1.40) or 40 × DIC (NA 1.3) oil immersion lenses. All cells were imaged at a resolution of 1024 × 1024 pixels. For neurons (Div > 8) that require a larger field of view to capture a single neuron, multiple images were taken under the 40 × lens first and mosaics of overlapping images were assembled in ImageJ (NIH, http://rsb.info.nih.gov/ij) using MosaicJ plugin [62].

### Confocal imaging of live and fixed cells for translocation experiment

Experiments to observe Rasal1 translocation were carried out as described in [63]. For live cell imaging of Rasal1 translocation, the hippocampal neurons were transfected with Rasal1-EGFP fusion protein using LipofectAMINE 2000 (Invitrogen) and were incubated with depolarizing solution containing 25 mM HEPES, pH 7.4, 90 mM NaCl, 40 mM KCl, 3 mM CaCl2, 1.4 mM MgCl2, and 10 mM glucose or basal medium (25 mM HEPES, pH 7.4, 140 mM NaCl, 4.7 mM KCl, 1.4 mM MgCl2, and 10 mM glucose) during live imaging.. EGFP fluorescence in cells was measured by confocal imaging at 1 Hz using a 63 × objective. To analyze the translocation process the relative increase in plasma membrane over cytosolic fluorescence intensity (R) was calculated with the relative plasma membrane translocation parameter [14].

### Image analyses

For analyzing the neurite length the neurons were traced semi-manually by both simple neurite tracer [64] and SynD [29]. Both program analyses showed the same trend and similar tracings. All neurons were traced by the researcher and again blind by a student at the lab. All graphs and tracings shown are from SynD. All synapse data were automatically analyzed by SynD.

### Coimmunoprecipitation

As described previously [61], the hippocampal neuron protein (300 μg) was incubated with Protein A/G Plus-Agarose beads (Santa Cruz Biotechnology, sc-2003) pretreated with Rasal1 antibody GenScript at 4 °C for 1 h. The cell lysates were centrifuged at 10,000 × *g* for 3 min. The supernatants were collected and incubated again with Protein A/G Plus-Agarose beads at 4 °C for 2 h, and centrifuged at 10,000 × *g* for 1 min. A sample of supernatant was collected to detect level of the unbound proteins using western blotting. The pellet was washed with RIPA buffer 4 °C for 5 min and centrifuged at 500 X g for 1 min, this step was repeated five times. The remaining pellet was mixed with 40 μL of Laemmli buffer (Bio-Rad; with 50 mM DTT). After vortexing, the mixture was boiled for 5 min, and centrifuged at 10,000 × *g* for 3 min. The supernatant was collected for western blot analysis.

### Protein affinity purification (pull-down)

For affinity pull-down experiments, three GST/Rasal1 fusion proteins, Rasal1-N-GST, Rasal1-M-GST, Rasal1-C-GST were prepared as described previously [65]. In all cases, GST was located at the N- or C-terminal end of Rasal1. Rasal1-N-GST contains the N-terminus end, Rasal1-M-GST the middle, and Rasal1-C-GST the C-terminus end. Specifically, the Rasal1 cDNA was amplified by PCR and cloned into pGEX4T-1. The constructs were resequenced to confirm appropriate insertion sites and the absence of spurious PCR generated nucleotide errors. Expression of the transformed plasmid in BL21 (DE3) Bacteria (C2527I; New England Biolabs) was induced by IPTG (isopropyl beta-D-1thiogalactopyranoside) (I5502; Sigma-Aldrich). GST-fusion protein was collected from bacterial lysate and purified using glutathione-Sepharose 4B beads as described by the manufacturer (17-0756-01; GE Healthcare).

For affinity purification experiments, the solubilized protein extracts (300 μg of protein) were incubated overnight at 4 °C with glutathione-Sepharose beads (GE Healtcare) bound to the indicated GST-fusion proteins (50 μg). Beads were washed three times with 600 μl of PBS containing 0.2% Triton X-100, and bound proteins were eluted with glutathione elution buffer. Eluates were incubated in sample buffer and subjected to 10% SDS-PAGE for Western blot analysis.

### Ca^2+^ imaging

To measure intracellular calcium ([Ca^2+^]_i_) levels, neurons were loaded with 2 µM Fura-2 AM (Molecular Probes, Eugene, OR, U.S.A.) in extracellular solution composed of (mM) 140 NaCl, 2 CaCl_2_, 1 MgCl_2_, 10 HEPES, 10 glucose, 4 KCl (pH 7.3–7.4 and 320–330 mOsm) for 30 min at 37 °C. Ratiometric imaging was performed by alternate excitation at 340 nm and 380 nm by a Deltaram V single monochromator (PTI) controlled by EasyRatioPro (PTI, Edison, NJ, U.S.A.) in a dark environment at room temperature [66]. The signals were recorded by an intensified charged-coupled device (ICCD) camera (PTI) and fluorescence intensity (Poenie-Tsien) ratios of images were calculated using EasyRatioPro. Only GFP positive cells were measured.

### Electrophysiological measurements

Whole-cell patch clamp recordings (ruptured) were performed on cultured hippocampal neurons using an Axopatch 200B patch-clamp amplifier and Digidata 1322A (Molecular Devices, Sunnyvale, CA, U.S.A.) digitizer, as described previously [66–68]. The external solution contained (mM) 120 NaCl, 5.4 KCl, 1 MgCl_2_, 2 CaCl_2_ 20 HEPES, and 10 glucose (pH 7.4, NaOH), as well as various blockers including 2 μM tetrodotoxin (TTX, Tocris), 200 μM CdCl_2_, 10 μM bicuculline (BCC, Sigma) and 20 μM CNQX (Tocris) to isolate spontaneous NMDA-mediated mEPSCs. The pipette (3–6 MΩ) was filled with the pipette solution containing (mM) 130 CsCl, 1 MgCl_2_, 2 Na_2_-ATP, 0.03 Na_2_-GTP, and 1 EGTA (pH 7.2, CsOH). A voltage ramp (from − 100 mV to 80 mV, over 300 ms) was applied with a holding potential of − 60 mV. The recordings were performed with or without 100 µM carbachol (CCh) at room temperature (22–25 °C). Clampfit 10.3 (Axon Instrument) and Origin 8.1 (OriginLab) were used for analyses of data.

### CaMKII phosphorylation assay

Cultured neurons were blocked with culture media supplemented with 1 µM TTX, 40 µM CNQX and 100 µM APV for 8 h. Blocked neurons were fixed and stained as normal after the 8 h incubation time. Activated neurons were stimulated with 10 µM glycine for 5 min before fixing with 4% PFA and 10 µM glycine, followed by immunostaining using the regular protocol.

### Quantification and statistical analysis

Statistical analysis was performed using GraphPad Prism software. Statistical significance was calculated using the two-tailed unpaired student t-test. Statistical significance was set at p < 0.05. All data are presented as mean ± SEM.

### Supplementary Information

**Additional file 1: Fig S1.** Manipulation of Rasal1 protein expression using p-Lenti-Rasal1-shRNA-EGFP and pAAV-Rasal1-FLAG constructs. **A** Representative confocal images of Rasal1 expression in EGFP-control, pAAV-Rasal1-FLAG or pLenti-Rasal1-shRNA-EGFP transduced hippocampal neurons stained for Rasal1 (red) and MAP2 (green). Scale bar: 20 µm. **B** Change in Rasal1 expression (as percent of control) of p-Lenti-Rasal1-shRNA-EGFPand pAAV-Rasal1-FLAG transduced hippocampal neurons. Data presented as Mean ± SEM. n = 60 cells in total per group. Experiment repeated 3 times. **C** Transfection efficiency of 30% in hippocampal neurons is shown with alpha-tubulin staining (cyan) as control and GFP expression in green. Anti-Flag staining for AAV constructs had a higher expression efficiency (data not shown). **D**, **E** Rasal1 expression in HEK293 cells transfected with a vector encoding mouse Rasal1-flag, (lanes 1–5), alone (lane 1) or in combination with four different shRNA constructs (lanes 2–5). As control the lower half of blot membrane was cut and stained against the household protein alpha-tubulin. The upper half was stained for the flag epitope. The strongest inhibition of Rasal1 protein levels was observed with shRNA construct #3. (Ratio Flag/alpha-tubulin = Rasal1-Flag: 1,38; shRNA#1: 1.01; shRNA#2: 1,39; shRNA#3: 0,76; shRNA#4: 1,36) **F** Rasal1 protein levels in primary hippocampal cultures transduced with p-Lenti-Rasal1-shRNA-EGFP #3, and 2 pAAV-Rasal1-FLAG over-expression constructs (OE1 and OE2) compared to controls determined using Western blot analysis. **G** Quantization of Western blot band intensities primary hippocampal cells transduced with p-Lenti-Rasal1-shRNA-EGFP #3, and 2 pAAV-Rasal1-FLAG over-expression constructs (OE1 and OE2) compared to controls (ratio Rasal1/Actin: control 0.154 ± 0.025; Rasal1-shRNA3: 0.033 ± 0.005; pAAV-Rasal1-Flag2-IRES-GFP: 2.9 ± 0.3; pLenti-Rasal1-Flag1: 1.4 ± 0.7).

**Additional file 2: Fig S2.** Design of Rasal1 shRNA vectors. Oligonucleotides and shRNA target sequences (bold) for Rasal1 knockdown. The leading T is required at the -1 position of the U6 promoter. The loop sequence is based on Brummelkamp et al., Science 2002.

## Data Availability

Not applicable.

## References

[1] Allen M, Chu S, Brill S, Stotler C, Buckler A (1998). Restricted tissue expression pattern of a novel human rasGAP-related gene and its murine ortholog. Gene.

[2] Bottomley JR, Reynolds JS, Lockyer PJ, Cullen PJ (1998). Structural and functional analysis of the putative inositol 1,3,4,5-tetrakisphosphate receptors GAP1IP4BPand GAP1m. Biochem Biophys Res Commun.

[3] Kupzig S, Deaconescu D, Bouyoucef D, Walker SA, Liu Q, Polte CL (2006). GAP1 family members constitute bifunctional Ras and Rap GTPase-activating proteins. J Biol Chem.

[4] Jin H, Wang X, Ying J, Wong AHY, Cui Y, Srivastava G (2007). Epigenetic silencing of a Ca(2+)-regulated Ras GTPase-activating protein RASAL defines a new mechanism of Ras activation in human cancers. Proc Natl Acad Sci U S A..

[5] Calvisi DF, Ladu S, Conner EA, Seo D, Hsieh J-T, Factor VM (2011). Inactivation of Ras GTPase-activating proteins promotes unrestrained activity of wild-type Ras in human liver cancer. J Hepatol.

[6] Qiao F, Su X, Qiu X, Qian D, Peng X, Chen H (2012). Enforced expression of RASAL1 suppresses cell proliferation and the transformation ability of gastric cancer cells. Oncol Rep.

[7] Bernards A, Settleman J (2004). GAP control: regulating the regulators of small GTPases. Trends Cell Biol.

[8] Nalefski EA, Falke JJ (1996). The C2 domain calcium-binding motif: structural and functional diversity. Protein Sci.

[9] Rizo J, Südhof TC (1998). C2-domains, structure and function of a universal Ca2+-binding domain. J Biol Chem.

[10] Cho W (2001). Membrane targeting by C1 and C2 domains. J Biol Chem.

[11] Sot B, Behrmann E, Raunser S, Wittinghofer A (2013). Ras GTPase activating (RasGAP) activity of the dual specificity GAP protein Rasal requires colocalization and C2 domain binding to lipid membranes. Proc Natl Acad Sci U S A..

[12] Liu Q, Walker SA, Gao D, Taylor JA, Dai Y-F, Arkell RS (2005). CAPRI and RASAL impose different modes of information processing on Ras due to contrasting temporal filtering of Ca2+. J Cell Biol.

[13] Lemmon MA (2003). Phosphoinositide recognition domains. Traffic.

[14] Walker SA, Kupzig S, Bouyoucef D, Davies LC, Tsuboi T, Bivona TG (2004). Identification of a Ras GTPase-activating protein regulated by receptor-mediated Ca2+ oscillations. EMBO J.

[15] Mattson MP, Kater SB. Calcium regulation of neurite elongation and growth cone motility. J Neurosci. 1987;7:4034 LP–4043. 10.1523/JNEUROSCI.07-12-04034.1987 PMC6569087 3121806

[16] Mattson MP, Murain M, Guthrie PB (1990). Localized calcium influx orients axon formation in embryonic hippocampal pyramidal neurons. Dev Brain Res.

[17] De Koninck P, Schulman H (1998). Sensitivity of CaM kinase II to the frequency of Ca2+ oscillations. Science.

[18] Chang HY, Takei K, Sydor AM, Born T, Rusnak F, Jay DG (1995). Asymmetric retraction of growth cone filopodia following focal inactivation of calcineurin. Nature.

[19] Lautermilch NJ, Spitzer NC (2000). Regulation of calcineurin by growth cone calcium waves controls neurite extension. J Neurosci.

[20] Xiang Y, Li Y, Zhang Z, Cui K, Wang S, Yuan X (2002). Nerve growth cone guidance mediated by G protein–coupled receptors. Nat Neurosci.

[21] Adams JP, Sweatt JD (2002). Molecular psychology: roles for the ERK MAP kinase cascade in memory. Annu Rev Pharmacol Toxicol.

[22] Sharma SK, Sherff CM, Shobe J, Bagnall MW, Sutton MA, Carew TJ (2003). Differential role of mitogen-activated protein kinase in three distinct phases of memory for sensitization in Aplysia. J Neurosci.

[23] Wadsworth P (1999). Regional regulation of microtubule dynamics in polarized, motile cells. Cell Motil.

[24] Robbins DJ, Zhen E, Cheng M, Xu S, Ebert D, Cobb MH. Map Kinases Erk1 And Erk2: pleiotropic enzymes in a ubiquitous signaling network. In: Vande Woude GF, Klein GBT-A in CR, editors. Academic Press; 1994. p. 93–116. 10.1016/s0065-230x(08)60399-1 8036991

[25] Witte H, Neukirchen D, Bradke F (2008). Microtubule stabilization specifies initial neuronal polarization. J Cell Biol.

[26] Eickholt BJ, Ahmed AI, Davies M, Papakonstanti EA, Pearce W, Starkey ML (2007). Control of axonal growth and regeneration of sensory neurons by the p110δ PI 3-Kinase. PLoS ONE.

[27] McCormick F (1998). Going for the GAP. Curr Biol.

[28] Iwashita S, Kobayashi M, Kubo Y, Hinohara Y, Sezaki M, Nakamura K (2007). Versatile roles of R-Ras GAP in neurite formation of PC12 cells and embryonic vascular development*. J Biol Chem.

[29] Schmitz SK, Hjorth JJJ, Joemai RMS, Wijntjes R, Eijgenraam S, de Bruijn P (2011). Automated analysis of neuronal morphology, synapse number and synaptic recruitment. J Neurosci Methods.

[30] Mitchison T, Kirschner M (1984). Dynamic instability of microtubule growth. Nature.

[31] Moutin M-J, Bosc C, Peris L, Andrieux A (2021). Tubulin post-translational modifications control neuronal development and functions. Dev Neurobiol.

[32] Kollins KM, Bell RL, Butts M, Withers GS (2009). Dendrites differ from axons in patterns of microtubule stability and polymerization during development. Neural Dev.

[33] Kim JH, Lee H-K, Takamiya K, Huganir RL. The Role of Synaptic GTPase-Activating Protein in Neuronal Development and Synaptic Plasticity. J Neurosci. 2003;23:1119 LP–1124. 10.1523/JNEUROSCI.23-04-01119.2003 PMC6742247 12598599

[34] Aamodt SM, Constantine-Paton M (1999). The role of neural activity in synaptic development and its implications for adult brain function. Adv Neurol.

[35] Ryan TA, Smith SJ, Reuter H (1996). The timing of synaptic vesicle endocytosis. Proc Natl Acad Sci U S A.

[36] Wloga D, Joachimiak E, Fabczak H (2017). Tubulin post-translational modifications and microtubule dynamics. Int J Mol Sci.

[37] Gundersen GG, Khawaja S, Bulinski JC (1987). Postpolymerization detyrosination of alpha-tubulin: a mechanism for subcellular differentiation of microtubules. J Cell Biol.

[38] Webster DR, Gundersen GG, Bulinski JC, Borisy GG (1987). Assembly and turnover of detyrosinated tubulin in vivo. J Cell Biol.

[39] Marcos S, Moreau J, Backer S, Job D, Andrieux A, Bloch-Gallego E (2009). Tubulin tyrosination is required for the proper organization and pathfinding of the growth cone. PLoS ONE.

[40] Schmidt M, Goebeler M, Posern G, Feller SM, Seitz CS, Bröcker E-B (2000). Ras-independent activation of the Raf/MEK/ERK pathway upon calcium-induced differentiation of keratinocytes. J Biol Chem.

[41] Yasuda R, Harvey CD, Zhong H, Sobczyk A, van Aelst L, Svoboda K (2006). Supersensitive Ras activation in dendrites and spines revealed by two-photon fluorescence lifetime imaging. Nat Neurosci.

[42] Henley JR, Huang K, Wang D, Poo M (2004). Calcium mediates bidirectional growth cone turning induced by myelin-associated glycoprotein. Neuron.

[43] Gomez TM, Spitzer NC (2000). Regulation of growth cone behavior by calcium: new dynamics to earlier perspectives. J Neurobiol.

[44] Nishiyama M, Hoshino A, Tsai L, Henley JR, Goshima Y, Tessier-Lavigne M (2003). Cyclic AMP/GMP-dependent modulation of Ca2+ channels sets the polarity of nerve growth-cone turning. Nature.

[45] Dent EW, Gertler FB (2003). Cytoskeletal dynamics and transport in growth cone motility and axon guidance. Neuron.

[46] Dehmelt L, Halpain S (2004). Actin and microtubules in neurite initiation: are MAPs the missing link?. J Neurobiol.

[47] Chen N, Furuya S, Doi H, Hashimoto Y, Kudo Y, Higashi H (2003). Ganglioside/calmodulin kinase ii signal inducing cdc42-mediated neuronal actin reorganization. Neuroscience.

[48] Giese KP, Fedorov NB, Filipkowski RK, Silva AJ. Autophosphorylation at Thr286 of the α calcium-calmodulin kinase II in LTP and learning. Science (1979). 1998;279:870 LP–873. 10.1126/science.279.5352.870 9452388

[49] Bayer K-U, De Koninck P, Leonard AS, Hell JW, Schulman H (2001). Interaction with the NMDA receptor locks CaMKII in an active conformation. Nature.

[50] Strack S, Choi S, Lovinger DM, Colbran RJ (1997). Translocation of autophosphorylated calcium/calmodulin-dependent protein kinase II to the postsynaptic density. J Biol Chem.

[51] Araki Y, Zeng M, Zhang M, Huganir RL (2015). Rapid dispersion of SynGAP from synaptic spines triggers AMPA receptor insertion and spine enlargement during LTP. Neuron.

[52] Zhu JJ, Qin Y, Zhao M, Van Aelst L, Malinow R (2002). Ras and rap control AMPA receptor trafficking during synaptic plasticity. Cell.

[53] Kennedy MB, Beale HC, Carlisle HJ, Washburn LR (2005). Integration of biochemical signalling in spines. Nat Rev Neurosci.

[54] Thomas GM, Huganir RL (2004). MAPK cascade signalling and synaptic plasticity. Nat Rev Neurosci.

[55] Lan J, Skeberdis VA, Jover T, Grooms SY, Lin Y, Araneda RC (2001). Protein kinase C modulates NMDA receptor trafficking and gating. Nat Neurosci.

[56] Wu M, Fang K, Wang W, Lin W, Guo L, Wang J (2019). Identification of key genes and pathways for Alzheimer’s disease via combined analysis of genome-wide expression profiling in the hippocampus. Biophys Rep.

[57] Sap KA, Guler AT, Bury A, Dekkers D, Demmers JAA, Reits EA (2021). Identification of full-length wild-type and mutant huntingtin interacting proteins by crosslinking immunoprecipitation in mice brain cortex. J Huntingtons Dis.

[58] Teeple E, Joshi P, Pande R, Huang Y, Karambe A, Latta-Mahieu M, et al. Single Nuclei sequencing of human putamen oligodendrocytes reveals altered heterogeneity and disease-associated changes in parkinson’s disease and multiple system atrophy. bioRxiv. 2021;2021.05.06.442967.

[59] Broeke JHP, Roelandse M, Luteijn MJ, Boiko T, Matus A, Toonen RF (2010). Munc18 and Munc13 regulate early neurite outgrowth. Biol Cell.

[60] Fath T, Ke YD, Gunning P, Götz J, Ittner LM (2009). Primary support cultures of hippocampal and substantia nigra neurons. Nat Protoc.

[61] Nejatbakhsh N, Guo C-H, Lu TZ, Pei L, Smit AB, Sun H-S (2011). Caltubin, a novel molluscan tubulin-interacting protein, promotes axonal growth and attenuates axonal degeneration of rodent neurons. J Neurosci..

[62] Thévenaz P, Unser M (2007). User-friendly semiautomated assembly of accurate image mosaics in microscopy. Microsc Res Tech.

[63] Groffen S, Brian E, Dudok J, Kampmeijer J, Toonen R, Verhage M (2004). Ca2+-induced recruitment of the secretory vesicle protein DOC2B to the target membrane. J Biol Chem.

[64] Longair MH, Baker DA, Armstrong JD (2011). Simple Neurite Tracer: open source software for reconstruction, visualization and analysis of neuronal processes. Bioinformatics.

[65] Jarvis SE, Barr W, Feng Z-P, Hamid J, Zamponi GW (2002). Molecular determinants of syntaxin 1 modulation of N-type calcium channels. J Biol Chem.

[66] Gomez TM, Zheng JQ (2006). The molecular basis for calcium-dependent axon pathfinding. Nat Rev Neurosci.

[67] Sun J, Pang ZP, Qin D, Fahim AT, Adachi R, Südhof TC (2007). A dual-Ca2+-sensor model for neurotransmitter release in a central synapse. Nature.

[68] Hui K, Fei G-H, Saab BJ, Su J, Roder JC, Feng Z-P (2007). Neuronal calcium sensor-1 modulation of optimal calcium level for neurite outgrowth. Development.

