# Supplementary figures and images for: Rasal1 regulates calcium dependent neuronal maturation by modifying microtubule dynamics
Source: Cell Biosci. 2024 Jan 21;14:13. doi: 10.1186/s13578-024-01193-w (PMC10800070; doi:10.1186/s13578-024-01193-w)

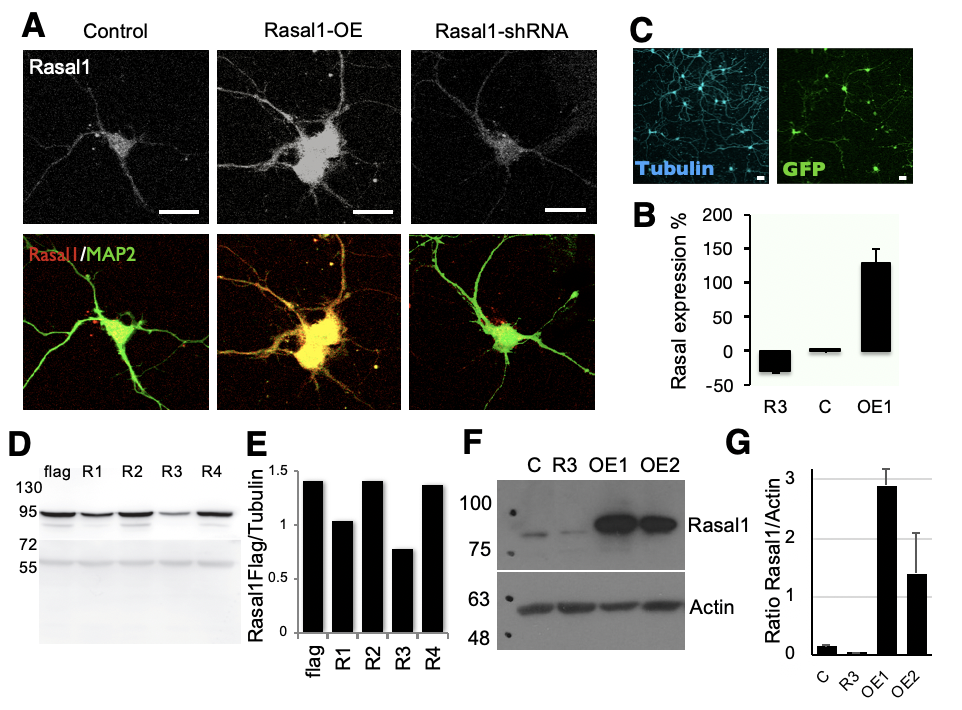

Supplement: Supplementary file 1 — Additional file 1: Fig S1. Manipulation of Rasal1 protein expression using p-Lenti-Rasal1-shRNA-EGFP and pAAV-Rasal1-FLAG constructs. A Representative confocal images of Rasal1 expression in EGFP-control, pAAV-Rasal1-FLAG or pLenti-Rasal1-shRNA-EGFP transduced hippocampal neurons stained for Rasal1 (red) and MAP2 (green). Scale bar: 20 µm. B Change in Rasal1 expression (as percent of control) of p-Lenti-Rasal1-shRNA-EGFPand pAAV-Rasal1-FLAG transduced hippocampal neurons. Data presented as Mean ± SEM. n = 60 cells in total per group. Experiment repeated 3 times. C Transfection efficiency of 30% in hippocampal neurons is shown with alpha-tubulin staining (cyan) as control and GFP expression in green. Anti-Flag staining for AAV constructs had a higher expression efficiency (data not shown). D, E Rasal1 expression in HEK293 cells transfected with a vector encoding mouse Rasal1-flag, (lanes 1–5), alone (lane 1) or in combination with four different shRNA constructs (lanes 2–5). As control the lower half of blot membrane was cut and stained against the household protein alpha-tubulin. The upper half was stained for the flag epitope. The strongest inhibition of Rasal1 protein levels was observed with shRNA construct #3. (Ratio Flag/alpha-tubulin = Rasal1-Flag: 1,38; shRNA#1: 1.01; shRNA#2: 1,39; shRNA#3: 0,76; shRNA#4: 1,36) F Rasal1 protein levels in primary hippocampal cultures transduced with p-Lenti-Rasal1-shRNA-EGFP #3, and 2 pAAV-Rasal1-FLAG over-expression constructs (OE1 and OE2) compared to controls determined using Western blot analysis. G Quantization of Western blot band intensities primary hippocampal cells transduced with p-Lenti-Rasal1-shRNA-EGFP #3, and 2 pAAV-Rasal1-FLAG over-expression constructs (OE1 and OE2) compared to controls (ratio Rasal1/Actin: control 0.154 ± 0.025; Rasal1-shRNA3: 0.033 ± 0.005; pAAV-Rasal1-Flag2-IRES-GFP: 2.9 ± 0.3; pLenti-Rasal1-Flag1: 1.4 ± 0.7). [file 13578_2024_1193_MOESM1_ESM.tiff]

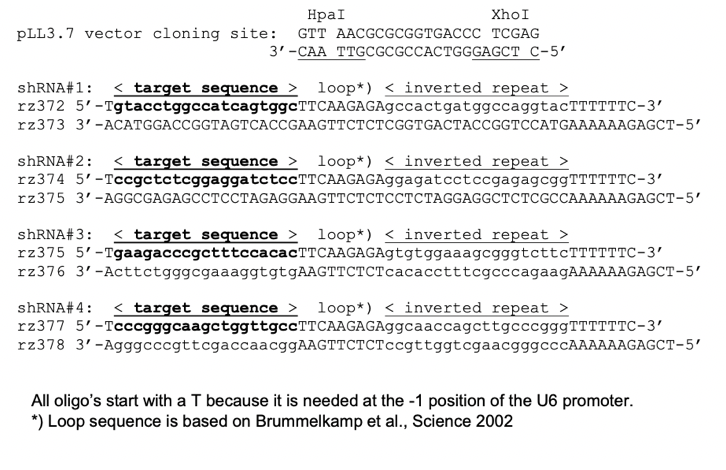

Supplement: Supplementary file 2 — Additional file 2: Fig S2. Design of Rasal1 shRNA vectors. Oligonucleotides and shRNA target sequences (bold) for Rasal1 knockdown. The leading T is required at the -1 position of the U6 promoter. The loop sequence is based on Brummelkamp et al., Science 2002. [file 13578_2024_1193_MOESM2_ESM.tiff]
